# Hydrogen Production and Li-Ion Battery Performance with MoS_2_-SiNWs-SWNTs@ZnONPs Nanocomposites

**DOI:** 10.3390/nano14231911

**Published:** 2024-11-28

**Authors:** Abniel Machín, María C. Cotto, Francisco Márquez, Jesús Díaz-Sánchez, Celia Polop, Carmen Morant

**Affiliations:** 1 Division of Natural Sciences and Technology, Universidad Ana G. Méndez-Cupey Campus, San Juan, PR 00926, USA; machina1@uagm.edu; 2 Nanomaterials Research Group, School of Natural Sciences and Technology, Universidad Ana G. Méndez-Gurabo Campus, Gurabo, PR 00778, USA; mcotto48@uagm.edu; 3 Department of Condensed Matter Physics, Universidad Autónoma de Madrid, 28049 Madrid, Spain; jesus.diazs@uam.es (J.D.-S.); celia.polop@uam.es (C.P.); 4 Instituto de Ciencia de Materiales Nicolás Cabrera, Universidad Autónoma de Madrid, 28049 Madrid, Spain; 5 Condensed Matter Physics Center (IFIMAC), Universidad Autónoma de Madrid, 28049 Madrid, Spain; 6 Department of Applied Physics, Universidad Autónoma de Madrid, 28049 Madrid, Spain

**Keywords:** Li-ion battery, hydrogen, HER, water splitting, photocatalysis

## Abstract

This study explores the hydrogen generation potential via water-splitting reactions under UV-vis radiation by using a synergistic assembly of ZnO nanoparticles integrated with MoS_2_, single-walled carbon nanotubes (SWNTs), and crystalline silicon nanowires (SiNWs) to create the MoS_2_-SiNWs-SWNTs@ZnONPs nanocomposites. A comparative analysis of MoS_2_ synthesized through chemical and physical exfoliation methods revealed that the chemically exfoliated MoS_2_ exhibited superior performance, thereby being selected for all subsequent measurements. The nanostructured materials demonstrated exceptional surface characteristics, with specific surface areas exceeding 300 m^2^ g^−1^. Notably, the hydrogen production rate achieved by a composite comprising 5% MoS_2_, 1.7% SiNWs, and 13.3% SWNTs at an 80% ZnONPs base was approximately 3909 µmol h^−1^g^−1^ under 500 nm wavelength radiation, marking a significant improvement of over 40-fold relative to pristine ZnONPs. This enhancement underscores the remarkable photocatalytic efficiency of the composites, maintaining high hydrogen production rates above 1500 µmol h^−1^g^−1^ even under radiation wavelengths exceeding 600 nm. Furthermore, the potential of these composites for energy storage and conversion applications, specifically within rechargeable lithium-ion batteries, was investigated. Composites, similar to those utilized for hydrogen production but excluding ZnONPs to address its limited theoretical capacity and electrical conductivity, were developed. The focus was on utilizing MoS_2_, SiNWs, and SWNTs as anode materials for Li-ion batteries. This strategic combination significantly improved the electronic conductivity and mechanical stability of the composite. Specifically, the composite with 56% MoS_2_, 24% SiNWs, and 20% SWNTs offered remarkable cyclic performance with high specific capacity values, achieving a complete stability of 1000 mA h g^−1^ after 100 cycles at 1 A g^−1^. These results illuminate the dual utility of the composites, not only as innovative catalysts for hydrogen production but also as advanced materials for energy storage technologies, showcasing their potential in contributing to sustainable energy solutions.

## 1. Introduction

In the face of a rapidly evolving global energy landscape, the transition to renewable and efficient energy sources has become imperative [1]. This shift is driven by the urgent need to combat climate change and reduce our reliance on fossil fuels [1,2]. Among the numerous strategies under exploration, hydrogen is emerging as a pivotal element in the future energy system due to its versatility, abundance, and potential to serve as a clean fuel with zero carbon emissions when produced through renewable processes such as water splitting [3]. As detailed in recent studies [4], hydrogen can play a critical role in decarbonizing hard-to-abate sectors such as transportation and industry. Its ability to store and release energy makes it a promising carrier for integrating intermittent renewable energy sources, such as solar and wind, into the grid. Furthermore, hydrogen can be utilized in fuel cells to power electric vehicles or be converted back to electricity, providing flexibility across multiple applications. This underscores the growing global focus on hydrogen production technologies that are both sustainable and efficient.

Advanced materials, particularly nanomaterials, have emerged as frontrunners in revolutionizing energy generation and storage technologies [3]. Carbon nanotubes (CNTs) [4,5], silicon nanowires (SiNWs) [6,7,8], and molybdenum disulfide (MoS_2_) [9] stand out due to their unique properties, which make them ideal candidates for enhancing both hydrogen production and lithium-ion battery (Li-ion) performance. This paper investigates the innovative integration of these nanomaterials into composites optimized for photocatalytic hydrogen generation [3,10,11,12,13,14,15,16,17], and energy storage [6,18,19,20,21,22,23], capitalizing on their synergistic properties to significantly boost efficiency.

Carbon nanotubes are well known for their exceptional mechanical strength and high electrical conductivity, properties that make them indispensable in energy applications [4,5]. In hydrogen production, CNTs enhance electron transport, improving the efficiency of photocatalytic water splitting [24]. Furthermore, CNTs provide robust structural support in battery electrodes, promoting durability and electron mobility—key factors for high-performance batteries [25]. Their role in these nanocomposites is to serve as a backbone, facilitating both electron flow and mechanical stability, which collectively improves the overall efficiency of the system [25,26]. Silicon nanowires, on the other hand, offer a high surface area-to-volume ratio, a feature that is advantageous for both catalytic and battery applications [27]. In photocatalysis, SiNWs expose a greater number of active sites for water-splitting reactions, thereby increasing hydrogen production rates [28]. For Li-ion batteries, this increased surface area facilitates enhanced interaction with the electrolyte, allowing for improved lithium-ion storage and retrieval [29]. This is critical for achieving high energy densities and faster charging times. MoS_2_, a transition metal dichalcogenide, is recognized for its outstanding catalytic properties, particularly in hydrogen evolution reactions (HER) [9,30]. Its layered structure hosts active sites that are pivotal in catalyzing hydrogen production from water. Beyond catalysis, MoS_2_ also contributes to the structural integrity and conductivity of battery electrodes when combined with other nanomaterials like CNTs and SiNWs [31].

In this study, we focus on the synthesis and performance of MoS_2_-SiNWs-SWNTs@ZnONPs nanocomposites, created through a strategic assembly that integrates zinc oxide nanoparticles (ZnONPs), MoS_2_, single-walled carbon nanotubes (SWNTs), and SiNWs. The selection of ZnONPs as a base material is driven by their excellent absorption properties, essential for initiating photocatalytic water splitting. We compare the performance of MoS_2_ synthesized through chemical and physical exfoliation, finding that the chemically exfoliated MoS_2_ exhibits superior catalytic activity. This formulation was then chosen for further testing. Photocatalytic tests of the nanocomposites under UV-visible light showed a marked increase in hydrogen production, with the optimized composite achieving a hydrogen generation rate of approximately 3909 µmol h^−1^g^−1^ under 500 nm wavelength radiation, more than 40 times higher than that of pristine ZnO nanoparticles. This significant enhancement is attributed to improved charge separation and increased catalytic activity due to the synergistic interactions among the nanocomposite components.

In addition to its application in hydrogen production, the potential of these nanocomposites for use in Li-ion batteries was also explored. Given the limitations of ZnO nanoparticles in terms of electrical conductivity and theoretical capacity, we developed a scalable synthesis process for MoS_2_ nanocomposites to be used as anode materials. Initial tests on pristine MoS_2_ electrodes revealed insufficient electrochemical performance. However, the integration of MoS_2_ nanosheets with CNTs in an 80:20 wt/wt ratio (MoS_2_@CNT) improved the performance significantly. Further enhancement was achieved by incorporating silicon nanowires into the MoS_2_-CNT matrix, forming a second series of free-standing electrodes (MoS_2_@SiNW@CNT) in a 56:24:20 wt.% composition. This composite exhibited a stable and homogeneous microstructure, free from agglomeration, as confirmed by energy-dispersive X-ray (EDX) mapping and field emission scanning electron microscopy (FESEM). The optimized electrode achieved a remarkable specific capacity of 1000 mAh g^−1^ after 100 cycles at a current density of 1 A g^−1^, demonstrating the potential of these nanocomposites to contribute to the development of high-capacity, long-life battery systems.

The choice of MoS_2_, SiNWs, and SWNTs for both hydrogen production and Li-ion battery applications is motivated by the unique multifunctional properties of these materials, which make them highly suitable for dual applications. MoS_2_, a well-known transition metal dichalcogenide, has exceptional catalytic properties in hydrogen evolution reactions (HER) due to its layered structure and sulfur vacancies that act as active sites for hydrogen production [20]. Simultaneously, MoS_2_’s ability to intercalate lithium ions within its layers makes it an excellent anode material for Li-ion batteries [25]. This dual capability stems from its two-dimensional structure, which allows for efficient charge separation in photocatalysis and effective lithium-ion storage in battery applications [30]. Thus, MoS_2_’s catalytic activity and its performance in energy storage provide a strong foundation for its use in both technologies.

SiNWs and SWNTs further enhance the performance of these nanocomposites by addressing critical limitations such as conductivity and structural stability. SiNWs offer a high surface area-to-volume ratio, providing more active sites for hydrogen production and improving interactions with electrolytes in Li-ion batteries, resulting in enhanced storage capacity [28]. Although silicon is known for its large volume expansion during lithiation, its nanowire structure helps mitigate this effect, preserving the integrity of the electrode over repeated charge–discharge cycles [15]. SWNTs contribute by acting as highly conductive pathways that facilitate electron transport and improve charge mobility [6]. Their mechanical strength also reinforces the structural stability of the composite, ensuring high performance in both catalytic and electrochemical systems [22]. Thus, the combination of these materials allows for the creation of multifunctional nanocomposites optimized for hydrogen production and energy storage.

The primary objective of this study was to demonstrate the dual functionality of the synthesized nanocomposites in advancing both hydrogen production and energy storage technologies. By harnessing the unique properties of each nanomaterial, this research highlights not only their individual contributions but also their combined potential to significantly improve the efficiency of energy systems. The comprehensive analysis of the photocatalytic and electrochemical performance of these nanocomposites underscores their promise as key components in sustainable energy solutions.

## 2. Materials and Methods

### 2.1. Materials

The solutions used in the synthesis were prepared with ultrapure deionized water (Milli-Q water) having a resistivity of 18.2 MΩ·cm at 25 °C to ensure high quality and consistency in experimental results. Zinc oxide nanoparticles (ZnONPs) were synthesized using zinc acetate dihydrate (Zn(C_2_H_3_O_2_)_2_·2H_2_O, 98.99% purity) and sodium hydroxide (NaOH, 99.0% purity), both sourced from Sigma Aldrich (Milwaukee, WI, USA). Molybdenum disulfide (MoS_2_), with 99% purity and an average particle size of 90 nm in nanopowder form (Sigma Aldrich, St. Louis, MO, USA), was later exfoliated as described in subsequent sections. Silicon nanowires (SiNWs) were obtained from Floatech S.L. (Madrid, Spain) and used as received. Ethylenediaminetetraacetic acid disodium salt (EDTA-Na_2_, ACS reagent grade, 99.4–100.6% purity) in powder form was also employed, along with sodium sulfide nonahydrate (Na_2_S·9H_2_O, >99.99% purity), sodium sulfite (Na_2_SO_3_, Puriss. p.a. Ph. Eur. grade, anhydrous, 98–100% purity), and sodium sulfate (Na_2_SO_4_, anhydrous, ACS reagent, >99.99% purity), all obtained from Sigma Aldrich. Nafion (5% wt solution, Grade D520CS) was sourced from Chemours Company (Wilmington, DE, USA). High-purity isopropanol (>99.9% for HPLC) and absolute ethanol (200 Proof, >99.5% purity, HPLC/Spectrophotometric grade) were both supplied by Sigma Aldrich. Ethylenediamine (EDA), purified by redistillation to >99.5% purity, and N,N-dimethylformamide (DMF, for HPLC, >99.9% purity) were also provided by Sigma Aldrich.

Single-walled carbon nanotubes (SWNTs) in aqueous suspension (0.2 wt.%, without surfactants) were used, supplied by TUBALL™ BATT (Luxembourg). For the preparation of Li-ion battery electrodes, additional materials were used, including a polyvinylidene fluoride (PVDF) membrane (Celgard 3501, PP-coated, Charlotte, NC, USA) and a 1M solution of LiPF_6_ electrolyte in a 1:1:1 mixture of ethylene carbonate (EC), diethyl carbonate (DEC), and dimethyl carbonate (DMC), also from Sigma Aldrich. Glass microfiber filter disks (Whatman grade GF/B, thickness 0.68 mm) used as spacers were likewise provided by Sigma Aldrich.

### 2.2. Synthesis of the ZnONPs

The methodology for synthesizing zinc oxide nanoparticles (ZnONPs) is well documented in the previous literature [32]. The process begins with the preparation of a zinc acetate dihydrate solution, where 25 mL of a 0.2 M solution of Zn(C_2_H_3_O_2_)_2_·2H_2_O is thoroughly mixed with 50 mL of deionized water. This mixture is then heated to maintain a constant temperature of 60 °C. Once the target temperature is reached, 25 mL of a 4 M sodium hydroxide (NaOH) solution is gradually introduced into the mixture, drop by drop, at a rate of approximately 0.5 mL/min. Following the addition of the sodium hydroxide solution, the resulting mixture is maintained at 60 °C with continuous stirring for two hours to ensure complete reaction and homogeneity. After this period, heating is discontinued, and the solution is allowed to naturally cool to room temperature. Upon cooling, the precipitated solids are collected by centrifugation at 6000 rpm for 10 min. To purify the product, the collected solids undergo multiple washing cycles. Each cycle consists of resuspending the precipitate in deionized water and centrifuging it again at 6000 rpm for 10 min to remove residual impurities and any unreacted materials. This process is repeated five times until the wash waters reach a neutral pH, confirming the removal of excess reactants and byproducts. Finally, the washed and purified zinc oxide nanoparticles are collected and spread out to dry. The drying process is conducted in a controlled environment, with the material exposed to a constant temperature of 60 °C overnight. This step is crucial to remove any remaining moisture and to ensure the stability of the ZnONPs. The result is a dry, fine powder of zinc oxide nanoparticles, ready for subsequent experimental use.

### 2.3. Exfoliation of MoS_2_

The exfoliation of MoS_2_ was carried out following the method described by Ghorai et al. [33], with slight modifications, combining intercalation with ethylenediamine (EDA) and subsequent ultrasonication in an organic solvent. Initially, 0.5 g of bulk MoS_2_ powder was transferred to a 100-mL round-bottom flask. To this, 25 mL of EDA was added and the mixture was then subjected to magnetic stirring at room temperature. This stirring was maintained for 24 h to allow for thorough adsorption of the EDA molecules onto the MoS_2_ surface. Next, the mixture was centrifuged at 6000 rpm for 40 min to separate the unadsorbed EDA from the MoS_2_ particles. The supernatant, containing the excess EDA, was decanted and discarded. The precipitate, now containing EDA-intercalated MoS_2_, was redispersed in 40 mL of N,N-dimethylformamide (DMF) and the mixture was magnetically stirred for 3 h at room temperature. After that, the mixture was subjected to centrifugation at 6000 rpm for 30 min to remove any remaining unadsorbed EDA and excess solvent. The supernatant was discarded, and the purified EDA-intercalated MoS_2_ precipitate was collected. This process of redispersion in DMF and centrifugation was repeated twice. The purified EDA-intercalated MoS_2_ was then transferred to a 100-mL Erlenmeyer flask containing 50 mL of fresh DMF. The mixture was sonicated using a tip sonicator for 4 h at room temperature. The sonicator was operated at 500 W and 20 kHz frequency. To maintain the temperature below 30 °C throughout the sonication process, the sample was placed in a water bath, and ice was periodically added. Following sonication, the resulting suspension exhibited a greenish colloidal appearance, indicating successful exfoliation of MoS_2_ into few-layer or single-layer nanosheets. To isolate these exfoliated nanosheets, the suspension was centrifuged at 6000 rpm, and the supernatants containing the exfoliated MoS_2_ nanosheets were carefully collected and stored in clean glass vials for further use. The yield of this exfoliation process was low (approximately 5%), necessitating 15 repetitions to obtain a sufficient quantity of material for composite preparation. All synthesized batches were combined into a single vial and further homogenized with DMF to ensure that the MoS_2_ in all composites exhibited consistent characteristics.

### 2.4. Synthesis of Composites

The synthesis of MoS_2_-SiNWs-SWNTs@ZnONPs composites was carried out through a multi-step process as follows: Initially, 500 mg of ZnONPs was dispersed in 50 mL of deionized water and subjected to ultrasonication (in an ultrasonic bath) for 20 min at room temperature to ensure a homogeneous suspension. Simultaneously, the appropriate amounts of SWNTs and SiNWs were combined in a separate Erlenmeyer flask and dispersed in 20 mL of deionized water. This SWNTs/SiNWs mixture was also ultrasonicated for 10 min at room temperature to achieve uniform dispersion. Subsequently, this SWNTs/SiNWs mixture was added dropwise (approximately 1 mL/min) to the previously prepared ZnONPs solution under continuous magnetic stirring. The resulting composite suspension was maintained under magnetic stirring at room temperature for 1 h to facilitate interaction between the components. Following this, a predetermined quantity of pre-exfoliated MoS_2_ was gradually added to the mixture to ensure proper integration into the composite. The entire suspension was then stirred for an additional 2 h. Upon completion of stirring, the suspension was centrifuged at 6000 rpm for 15 min to separate the solid phase from the supernatant. The resulting precipitate was washed several times with deionized water to remove any unreacted species or residual contaminants. This washing process was coupled with two additional cycles of centrifugation to ensure the purity of the composite material. The final solid product was dried in a vacuum oven at 70 °C for 6 h to eliminate any remaining moisture. Subsequently, the dried material underwent thermal treatment at 300 °C using a temperature ramp of 5 °C/min. Once the temperature reached 300 °C, the treatment was maintained for 2 h under a continuous nitrogen flow (300 mL min^−1^) to enhance the stability of the composite. The composite material obtained after thermal treatment was carefully collected and stored in an airtight container for further characterization and potential application studies.

### 2.5. Characterization of Composites

The surface area of the composites was quantified using the Brunauer–Emmett–Teller (BET) method on a Micrometrics ASAP 2020 instrument, which employed nitrogen adsorption isotherms at 77 K (Micrometrics Instrument Corporation, Norcross, GA, USA). The morphology and structural details of the composite materials were examined through field emission scanning electron microscopy (FESEM) with an FEI Verios 460L system, integrated with a Quantax energy-dispersive X-ray spectroscopy (EDS) analyzer (Thermo Fisher Scientific, Hillsboro, OR, USA). High-resolution transmission electron microscopy (HRTEM) analysis was performed on a JEM 3000F microscope (JEOL, Peabody, MA, USA) to gain insights into the nanoscale features and lattice fringes of the samples. The crystalline phases present in the composites were determined using X-ray diffraction (XRD) analysis carried out with a Bruker D8 Advance diffractometer operating at 40 kV and 40 mA (Bruker Corporation, Billerica, MA, USA). Raman spectroscopy measurements were conducted with a DXR Raman microscope (Thermo Fisher Scientific, Waltham, MA, USA), utilizing a 532 nm laser at 5 mW power and a resolution of 5 cm^–1^, to investigate the vibrational modes of the materials. X-ray photoelectron spectroscopy (XPS) was employed to analyze the surface elemental composition and chemical states using an ESCALAB 220i-XL spectrometer with non-monochromatic magnesium Kα radiation (1253.6 eV) operating at 20 mA and 12 kV (Thermo Fisher Scientific, Waltham, MA, USA). The optical bandgap energies of the composites were obtained using a Perkin Elmer Lambda 1050 UV–Visible-NIR Spectrophotometer (Perkin Elmer, Waltham, MA, USA). Photoluminescence (PL) spectra were recorded to study the electronic properties using an FS900 Fluorescence Spectrometer (Edinburgh Instruments Ltd., Livingston, UK). The produced hydrogen was quantified by gas chromatography (GC) using a thermal conductivity detector (GC–TCD, Perkin-Elmer Clarus 600). The photoelectrochemical properties (transient photocurrent response) of the different materials were characterized using a CHI660D electrochemical system workstation (Shanghai Chenhua Instrument Co., Shanghai, China). Electrochemical measurements, including galvanostatic charge/discharge (GCD) curves, were conducted at room temperature using a 12-channel Arbin Instruments BT2143 workstation, with a potential window of 0.01 V to 3.0 V (vs. Li/Li^+^) at various current densities. Cyclic Voltammetry (CV) was performed using a Potentiostat/Galvanostat VersaSTAT 3 (Princeton Applied Research, Oak Ridge, TN, USA) at a scan rate of 0.2 mV/s over the same potential range of 0.01 V to 3.0 V. Electrochemical Impedance Spectroscopy (EIS) was also carried out with the PAR VersaSTAT 3, applying a 5 mV amplitude over a frequency range from 0.1 Hz to 1 MHz.

### 2.6. Photocatalytic Experiments

Optimal conditions for the catalytic hydrogen evolution reaction (HER) studies were determined in terms of catalyst dosage (0.5 g/L to 1.5 g/L), and pH levels (4 to 11) prior to initiating the experimental procedures. The photocatalytic hydrogen generation was evaluated by introducing 50 mg of the selected catalyst into a 200-mL quartz reactor flask containing 100 mL of deionized water. The reaction mixture was maintained at a constant temperature of 20 °C, agitated using a magnetic stirrer set at 20 rpm, and purged with nitrogen gas for a minimum of 20 min to remove any dissolved oxygen.

Subsequently, the solution was exposed to ultraviolet–visible (UV–vis) radiation for 120 min. Various filters were used to select the specific irradiation wavelengths appropriate for the study. To facilitate the reaction, solutions of 0.5 M sodium sulfide (Na_2_S) and 0.03 M sodium sulfite (Na_2_SO_3_) were added as sacrificial agents. The hydrogen produced during the reaction was carried by a nitrogen gas stream, collected, and subsequently analyzed. The evolved hydrogen was captured, and its volume quantitatively measured using a gas chromatographic system equipped with a thermal conductivity detector (GC-TCD). A Perkin Elmer Clarus 600 instrument was specifically used for this analysis.

### 2.7. Fabrication of Free-Standing MoS_2_ Based Electrodes

Three types of electrodes were prepared, each with varying proportions of MoS_2_ as the active material: pure MoS_2_, MoS_2_@CNTs (80:20 wt.%), and MoS_2_@SiNWs@CNTs (56:24:20 wt.%). The exfoliation of MoS_2_ followed the procedure previously described. The carbon nanotubes (CNTs) used had diameters ranging from 40 to 60 nm and lengths between 1 and 50 μm, with a purity exceeding 95%, as indicated by the supplier. The silicon nanowires (SiNWs) had diameters between 25 and 70 nm and straight lengths of 0.5 to 2 μm, as confirmed by field-emission scanning electron microscopy (FESEM). Some nanowires showed thicker diameters, up to 140 nm, and longer, curved lengths reaching 20 μm.

Freestanding electrodes were fabricated by preparing isopropanol (IPA) solutions with varying concentrations of MoS_2_ or a mixture of MoS_2_/SiNWs as the active material. These solutions were then poured into a funnel and filtered through a porous polyvinylidene fluoride (PVDF) membrane with a thickness of 25 μm, using positive pressure applied by a vacuum pump. Before preparing the composite, the CNT solutions in deionized water were sonicated using a probe sonicator for 20 min. New solutions were then prepared by mixing the sonicated CNTs with the corresponding active materials (MoS_2_ or MoS_2_@SiNWs) in IPA, ensuring the appropriate weight proportion of dried CNTs. After thorough mixing, the dispersion was sonicated again for 10 min at room temperature, leading to good entanglement between the CNTs and MoS_2_ (or MoS_2_@SiNWs), effectively preventing aggregate formation. All production steps were conducted under open-atmosphere conditions. Filtration was carried out immediately after sonication to prevent precipitate formation. This step is essential, as inhomogeneities from MoS_2_ agglomerates, SiNW clusters, or CNT bundles can disrupt network continuity and significantly affect battery performance. The amount of material used during filtration was standardized to 1 mg cm^−2^ of membrane area. After vacuum filtration, the compounds deposited on the PVDF membrane were placed in a desiccator for at least 2 h. The resulting electrodes, still attached to the membrane, were then cut into 12 mm discs and further dried under vacuum at 10^−^³ mbar and 90 °C for 10 h. Following this, the electrodes were transferred to an Ar-filled glovebox (GP Campus, Jacomex) with moisture and oxygen levels below 1.0 ppm, for battery assembly. The 2032 coin-type half-cells were assembled using pure lithium (Li) as the counter electrode. The electrolyte employed was a 1.0 M solution of LiPF_6_ in EC:DEC:DMC (1:1:1). A 16-mm-diameter glass microfiber filter disk was placed in direct contact with the PVDF membrane supporting the electrode, serving as both the separator and the support. After assembly, the cells were allowed to stabilize for a minimum of 4 h before measurement.

### 2.8. Postmortem Characterization of Li-Ion Coin Cells

After electrochemical cycling, selected coin cells were disassembled inside a glove box under an inert atmosphere using a precision non-conductor screwdriver. The lithium metal was carefully removed, and the electrode was extracted using fine-tipped tweezers. The electrode was subsequently cleaned with a dimethyl terephthalate (DMT) solution, followed by rinsing with an anhydrous solvent, and dried under controlled environmental conditions.

For postmortem characterization, the cycled electrodes were transferred with care and analyzed using scanning electron microscopy (SEM) and nuclear reaction analysis (NRA). NRA was employed to investigate lithium diffusion profiles within the cycled electrodes. The measurements were conducted using a tandem setup of high-current coaxial tandetron accelerators operating at a terminal voltage of 5 MV [34]. This technique, based on the nuclear reaction ^7^Li(p,α) ^4^He, is highly sensitive to the depth distribution of lithium atoms [35,36]. To optimize the NRA signal for lithium detection, 3 MeV H^+^ ions were used following the conditions established by Paneta et al. [37]. Backscattered ions were detected with two surface barrier detectors, having energy resolutions of 12 keV and 18 keV, positioned at scattering angles of 170° and 165° relative to the beam direction, respectively. The resulting NRA spectra were processed and analyzed using SIMNRA software, version 7.03 [38,39,40].

## 3. Results

### 3.1. Hydrogen Evolution Reaction (HER)

#### 3.1.1. Characterization of Composites

The synthesized nanomaterials were employed in the fabrication of photocatalysts, which were then tested for their efficiency in hydrogen production via water splitting (HER). Additionally, these nanomaterials were used to assemble electrodes for Li-ion batteries. The photocatalyst that demonstrated the highest efficiency in HER was 6.7% (MoS_2_-SiNWs)@ZnONPs-CNTs, making this material the focus of a thorough characterization.

The BET surface area of the different composites was measured, and the results are summarized in Table S1. The unmodified ZnONPs exhibited a surface area of 62 m^2^ g^−1^, which increased to approximately 297 m^2^ g^−1^ with the addition of CNTs and SiNWs. This increase in surface area is primarily attributed to the presence of CNTs in the composite. The introduction of exfoliated MoS_2_ (see Table S1) initially reduced the surface area to 231 m^2^ g^−1^ for the 5% (MoS_2_-SiNWs)@ZnONPs-CNTs catalyst and then slightly increased it to 246 m^2^ g^−1^ with a higher MoS_2_ content, as observed in the 6.7% (MoS_2_-SiNWs)@ZnONPs-CNTs catalyst. The observed trend in the specific surface area, where the value decreases and then slightly increases after adding MoS_2_ to the composite, can be attributed to the structural behavior of MoS_2_ and its interaction with other materials in the composite. Initially, the introduction of MoS_2_ leads to a decrease in surface area (from 297 m^2^ g^−1^ to 231 m^2^ g^−1^) due to the restacking of MoS_2_ layers. Exfoliated MoS_2_ nanosheets are known to exhibit van der Waals interactions, which cause aggregation and restacking, thereby reducing the accessible surface area. This is a common issue with layered transition metal dichalcogenides like MoS_2_, where inadequate exfoliation can diminish the porosity and surface area of the composite [41,42].

As the MoS_2_ content is increased, the surface area shows a slight recovery (to 246 m^2^ g^−1^), likely due to improved dispersion and interaction with other components in the composite, such as CNTs and ZnONPs. The higher concentration of MoS_2_ could promote better separation of nanosheets or create additional defects and interstitial spaces, thereby enhancing the overall surface area. These defects may contribute to a higher porosity, which counteracts the initial aggregation effect [43,44]. This balance between restacking at lower concentrations and improved dispersion at higher concentrations is characteristic of hybrid nanomaterial systems and emphasizes the importance of optimizing exfoliation processes in composite design.

**Figure 1:**
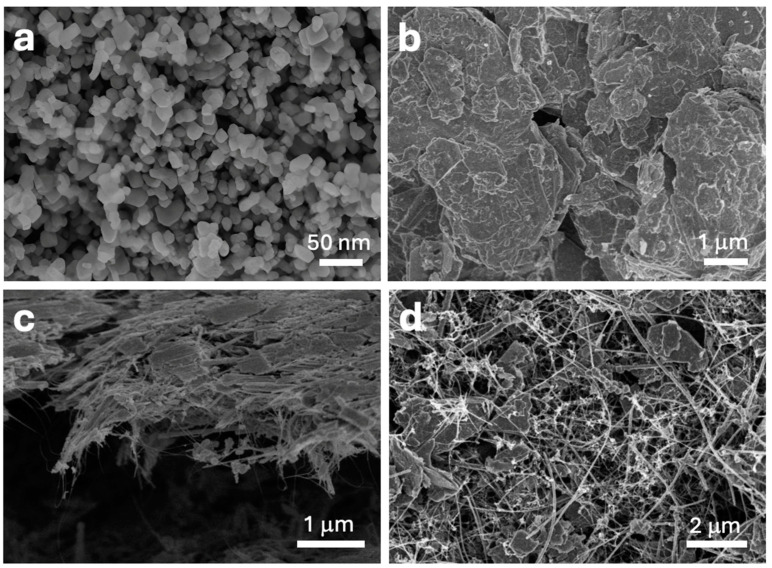
FESEM images showing ZnONPs (**a**); chemically exfoliated MoS_2_ (**b**); the adduct formed by MoS_2_ and CNTs (**c**); and the catalyst composed of ZnONPs, MoS_2_, CNTs, and SiNWs (**d**).

The catalyst precursors were thoroughly characterized using field emission scanning electron microscopy (FESEM), as shown in Figure 1. The ZnONPs (Figure 1a) exhibit heterogeneous particles with diameters ranging from approximately 15 to 25 nm. The chemically exfoliated MoS_2_ (Figure 1b) consists of small, thin sheets with highly variable sizes, ranging from 1 micron to several microns in average diameter. These sheets appear to be composed of multiple layers of MoS_2_; however, as will be discussed later in the high-resolution transmission electron microscopy (HRTEM) analysis, some of these sheets are actually single-layer MoS_2_. The variability in sheet thickness and lateral dimensions is expected to impact the electronic properties and catalytic activity of the material, as single-layer MoS_2_ typically exhibits distinct electronic characteristics compared to its multilayer counterparts. Figure 1c illustrates the adduct formed by MoS_2_ and carbon nanotubes (CNTs), revealing a highly homogeneous interweaving of both materials. This uniform integration is anticipated to be crucial for the catalytic performance, as the intimate contact between MoS_2_ and CNTs can enhance electron transport and catalytic efficiency. The well-dispersed CNT network provides mechanical support and electrical conductivity, which are likely to play a significant role in the overall activity and stability of the catalysts. Finally, Figure 1d displays the combination of all the materials—MoS_2_, CNTs, SiNWs, and ZnONPs. The image reveals a relatively homogeneous dispersion among all components, which is essential for ensuring consistent catalytic activity across the composite material. The uniform distribution of these components suggests effective mixing and interaction at the nanoscale, which may contribute to enhanced catalytic properties due to the synergistic effects of the individual materials.

**Figure 2:**
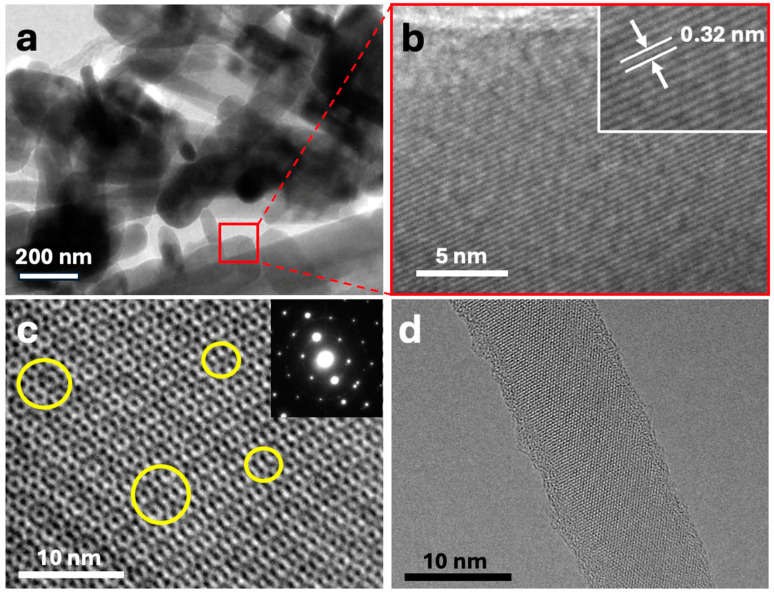
HRTEM images showing ZnO nanoparticles (ZnONPs) at different magnifications (**a**,**b**). The inset in (**b**) corresponds to a further magnified region, highlighting the lattice fringes. Chemically exfoliated MoS_2_ monolayer, with some regions displaying structural defects marked by yellow circles and SAED pattern (**c**), and the detailed image of a highly crystalline silicon nanowire (SiNW) (**d**).

The different components were further characterized using HRTEM. Figure 2a shows the HRTEM image of the ZnONPs, which exhibit heterogeneous dimensions, consistent with the observations made using FESEM. The nanoparticles display a range of sizes, reinforcing the earlier findings and suggesting a complex synthesis process that results in a non-uniform particle distribution. Figure 2b provides a magnified view of the highlighted region in Figure 2a, with an inset showing an even higher magnification where the material’s lattice fringes are clearly visible. The characteristic interplanar spacing of 0.32 nm observed in these fringes has been definitively attributed to the ZnO wurtzite structure [41], confirming the crystalline phase of the ZnONPs. Figure 2c illustrates an HRTEM image of a monolayer MoS_2_ sheet, where several defects can be observed (highlighted with yellow circles). These defects are hypothesized to contribute to the enhanced catalytic activity of the material, as defect sites can serve as active sites for catalytic reactions. Although the nature and exact influence of these defects are currently under investigation in a separate study, it is likely that they were introduced during the rigorous chemical exfoliation process that the material underwent. The presence of these defects may alter the electronic properties of the MoS_2_ monolayer, potentially leading to improved performance in catalytic applications [42]. Figure 2d shows a detailed HRTEM image of an SiNW, revealing its high crystallinity and a diameter of approximately 11–12 nm. The well-defined crystalline structure observed in the SiNW, which even reveals individual Si atoms, is indicative of a high-quality material, essential for applications where electronic properties are critical. The carbon nanotubes (CNTs) are not shown in the HRTEM images, as they are commercially sourced and have been extensively characterized. Given their well-documented properties, their detailed characterization was deemed unnecessary for this investigation.

**Figure 3:**
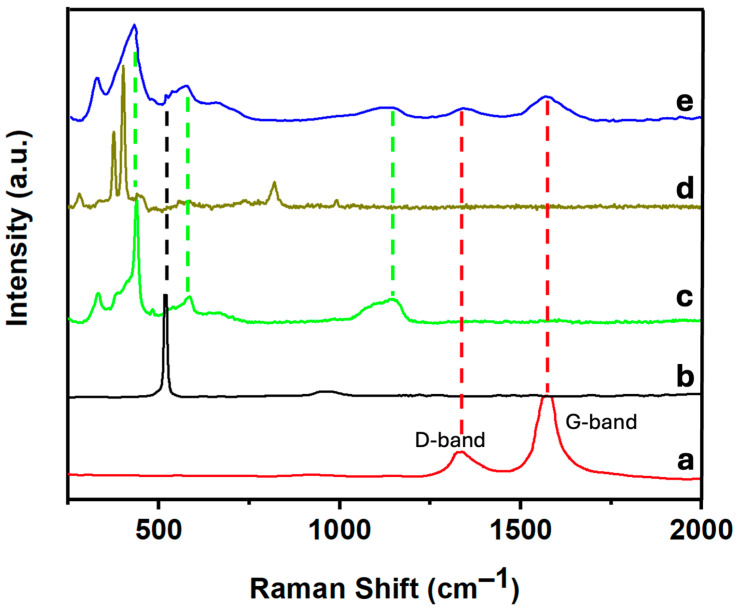
Raman spectra of CNTs (a); SiNWs (b); ZnONPs (c); MoS_2_ (d); and 6.7% (MoS_2_-SiNWs)@ZnONPs-CNTs (e).

Figure 3 shows the Raman spectra of CNTs, SiNWs, ZnONPs, MoS_2_, and the 6.7% (MoS_2_-SiNWs)@ZnONPs-CNTs catalyst. The CNTs (Figure 3a) exhibit two peaks at approximately 1338 cm^−1^ and 1576 cm^−1^, corresponding to the D band, which is associated with the presence of defects in the material, and the G band, respectively, both of which are characteristic of carbon nanotubes [43]. Figure 3b shows the Raman spectrum of the SiNWs, showing a characteristic peak at approximately 518 cm^−1^, which can be attributed to the first-order phonon mode [44]. The Raman spectrum of ZnONPs (Figure 3c) displays distinct peaks at ca. 327 cm^−1^, 437 cm^−1^, 560 cm^−1^, and a broad band around 1160 cm^−1^. The 327 cm^−1^ peak is attributed to the second-order Raman spectrum, while the 437 cm^−1^ peak is assigned to the E_2_ modes of Zn motion, corresponding to the characteristic band of the wurtzite phase [45,46]. The 560 cm^−1^ band is associated with the E_1_ mode, typically originating from second-order Raman scattering, and the broad band at 1160 cm^−1^ is attributed to overtones and/or combination bands [45,46,47]. The Raman spectrum of MoS_2_ (Figure 3d) shows two characteristic bands at approximately 384 cm^−1^ and 408 cm^−1^, which have been assigned to the E^1^_2_g and A_1_g modes, respectively [48]. These bands are indicative of the exfoliation process and the formation of MoS_2_ flakes with few layers [49,50]. The 6.7% (MoS_2_-SiNWs)@ZnONPs-CNTs catalyst (Figure 3e) exhibits peaks corresponding to the various components within the sample, albeit with differing intensities due to the proportions of the components in the catalyst. The detection of significant peaks from all catalyst components confirms the heterostructured nature of the composite.

**Figure 4:**
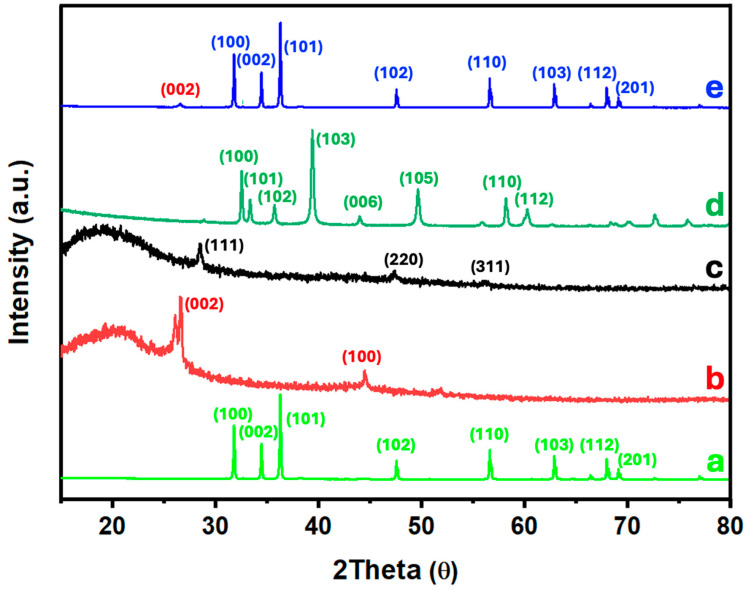
XRD patterns of ZnONPs (a); CNTs (b); SiNWs (c); MoS_2_ (d); and 6.7% (MoS_2_-SiNWs)@ZnONPs-CNTs (e).

Figure 4 shows the X-ray diffraction (XRD) patterns of the 6.7% (MoS_2_-SiNWs)@ZnONPs-CNTs catalyst, along with the individual XRD patterns of ZnONPs, CNTs, SiNWs, and MoS_2_ for comparison purposes. The diffraction peaks observed for ZnONPs (Figure 4a) can be clearly indexed to the hexagonal wurtzite ZnO phase, which dominates the diffraction pattern of the 6.7% (MoS_2_-SiNWs)@ZnONPs-CNTs catalyst, as shown in Figure 4e. The strong and distinct reflections are indicative of the crystalline nature of the ZnONPs and confirm their predominant presence within the composite catalyst [51]. The XRD pattern of CNTs (Figure 4b) reveals several peaks, which have been assigned to the (002) and (100) reflections characteristic of single-walled carbon nanotubes [52]. These reflections are consistent with the graphitic structure of CNTs, confirming their structural integrity within the composite. The diffraction pattern of SiNWs (Figure 4c) exhibits very weak intensities, characterized by three faint reflections assigned to the (111), (220), and (311) planes [53]. These weak reflections are typical of highly homogeneous and crystalline silicon nanowires, similar to those employed in this study. The low intensity of these peaks can be attributed to the small size and high dispersion of the SiNWs within the catalyst matrix. The MoS_2_ XRD pattern (Figure 4d) displays multiple diffraction peaks located at approximately 32°, 36°, 39°, 49°, and 58°, corresponding to the (100), (102), (103), (105), and (110) crystalline planes of the 2H-type MoS_2_ hexagonal phase, in accordance with the JCPDS # 75-1539 standard [48,54,55]. These reflections confirm the hexagonal crystalline structure of the MoS_2_ layers used in the catalyst preparation. In Figure 4e, the XRD pattern of the 6.7% (MoS_2_-SiNWs)@ZnONPs-CNTs catalyst is shown, highlighting the diffraction peaks of the composite’s constituents. To facilitate the identification of each component, a consistent color-coding scheme has been employed across the figures. The XRD pattern is dominated by the intense peaks from ZnONPs, the major component of the catalyst. Additionally, a minor peak at a low angle can be attributed to the presence of CNTs. The absence of discernible peaks from other components, such as MoS_2_ and SiNWs, in the catalyst’s XRD pattern can be explained by the high degree of dispersion and the relatively low proportions of these materials within the composite. This high dispersion effectively reduces the crystallite size to the point where the diffraction signals from these components are below the detection limit of the XRD technique, further emphasizing the successful integration and distribution of the various components in the catalyst structure.

**Figure 5:**
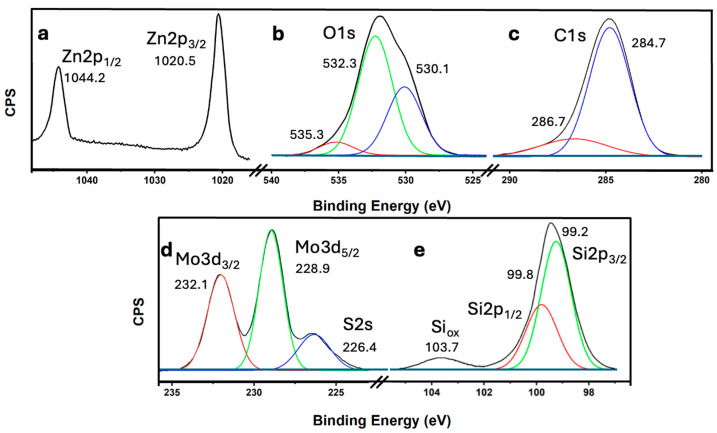
X-ray photoelectron spectroscopy (XPS) spectra of Zn 2p (**a**); O 1s (**b**); C1s (**c**); Mo3d (**d**) and Si2p (**e**).

The representative elements of the most efficient catalyst, 6.7% (MoS_2_-SiNWs)@ZnONPs-CNTs, were thoroughly characterized by X-ray photoelectron spectroscopy (XPS). The Zn2p spectrum (Figure 5a) reveals two distinct components at binding energies of 1044.2 eV and 1020.5 eV, corresponding to the Zn2p_1/2_ and Zn2p_3/2_ transitions of Zn^2+^, respectively [56]. The characteristic spin-orbit splitting of 23.7 eV confirms the oxidation state of Zn as Zn^2+^ [56]. Both transitions exhibit high symmetry, and attempts to fit the data to other possible zinc states yielded no significant results, thereby ruling out any additional contributions from other oxidation states. Figure 5b shows the O1s transition, which displays a clear asymmetry. This transition has been deconvoluted into three components at approximately 530.1, 532.3, and 535.3 eV. The peak at 530.1 eV is attributed to O^2−^ species within the ZnO lattice, indicating the presence of oxygen in a well-defined crystalline environment [57]. The component at 532.3 eV is assigned to O^2−^ in oxygen-deficient regions, suggesting the presence of defects or non-stoichiometric areas within the ZnO structure [57]. The highest binding energy peak at 535.3 eV likely corresponds to species generated by the interaction of ZnO nanoparticles with other components in the catalyst, possibly indicating surface modifications or interactions with other phases [57]. The C1s spectrum (Figure 5c) is also asymmetric and has been deconvoluted into two components at approximately 284.7 eV and 286.7 eV. The dominant peak at 284.7 eV is assigned to sp^2^-hybridized carbon, characteristic of the CNTs present in the catalyst [56,58]. The secondary peak at 286.7 eV is attributed to C-OH groups, likely arising from structural defects in the CNTs, which could be introduced during the synthesis or functionalization process [58]. The Mo3d and S2s transitions are presented in Figure 5d. The Mo3d spectrum features two well-defined and symmetrical peaks at 232.1 eV and 228.9 eV, corresponding to the Mo3d_3/2_ and Mo3d_5/2_ doublet, respectively [56]. These peaks are indicative of the Mo^4+^ state in MoS_2_, confirming the presence of MoS_2_ as a component of the catalyst [59,60]. Additionally, a peak observed at approximately 226.4 eV is attributed to the S2s transition, further confirming the presence of MoS_2_ within the catalyst [59]. The Si2p transition, as shown in Figure 5e, exhibits two deconvoluted peaks. The most intense peak, observed below 100 eV, is clearly asymmetric and was deconvoluted into two peaks at 99.8 eV and 99.2 eV. These peaks correspond to the Si2p_1/2_ and Si2p_3/2_ transitions of elemental silicon (Si) [56], with a characteristic spin-orbit coupling of approximately 0.6 eV. Additionally, a less intense peak at 103.7 eV is assigned to oxidized silicon (SiOx) [56], which likely originates from the surface oxidation of the silicon nanowires (SiNWs). This oxidation could be a result of exposure to ambient conditions or interactions with other components within the catalyst.

**Figure 6:**
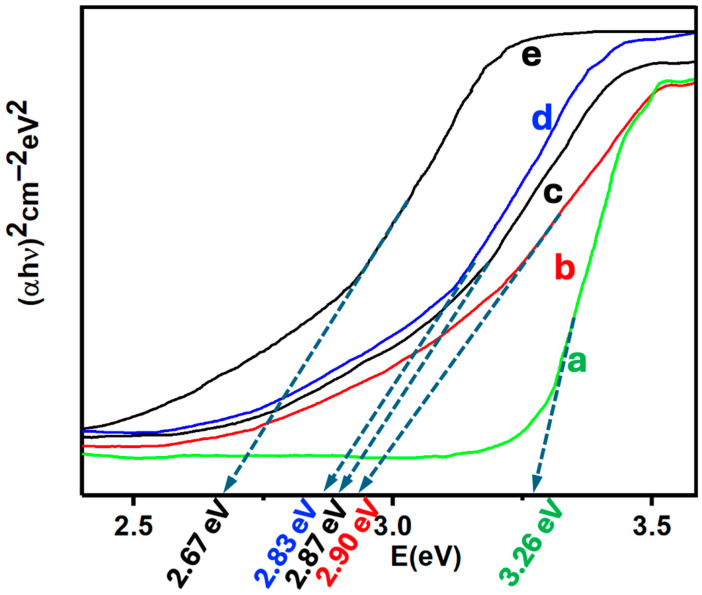
Tauc plots of (αhn)^2^ versus energy (eV), and determination of the bandgap energy of ZnONPs (a); ZnONPs-CNTs (b); 5% (MoS_2_-SiNWs)@ZnONPs-CNTs (c); 6.7% (MoS_2_-SiNWs)@ZnONPs-CNTs (d); and MoS_2_-SiNWs (e).

Diffuse reflectance spectroscopy was used to characterize both the precursors and the catalysts. The reflectance data, expressed in Kubelka-Munk units, enabled the determination of bandgaps via Tauc plots (see Figure 6). The bandgap energy of the wurtzite phase of ZnO is typically around 3.37 eV [61], which corresponds to its well-known ultraviolet absorption properties. However, the synthesized ZnO nanoparticles (ZnONPs) exhibited a slightly lower bandgap of 3.26 eV, likely due to variations in morphology, particle size, and the quantum confinement effect, all of which influence the semiconductor’s electronic properties. Smaller particle sizes, for example, can increase the surface-to-volume ratio and induce quantum confinement, shifting the bandgap lower than the bulk material [62]. This slight reduction in bandgap can enhance the material’s interaction with visible light, although it remains predominantly active in the ultraviolet region.

Incorporating carbon nanotubes (CNTs) into the ZnONPs further reduced the bandgap to 2.90 eV (Figure 6b). This significant reduction can be attributed to the electronic interaction between ZnONPs and CNTs, which results in the formation of new electronic states and the alteration of the band structure at the interface of the two materials [62]. CNTs, with their excellent electrical conductivity and large surface area, enhance the electron transport properties of the composite, improving the efficiency of photocatalytic processes under visible light. This bandgap shift into the visible region allows the composite to utilize a broader range of the solar spectrum, improving its overall photocatalytic efficiency.

MoS_2_, with a bandgap of approximately 2.5 eV [63], is also known for its ability to shift the bandgap towards the visible region when incorporated into heterostructures. The combination of MoS_2_ with ZnONPs and CNTs forms a heterojunction, which facilitates efficient charge separation by creating built-in electric fields at the interfaces, further improving the catalytic performance of the composite. Silicon nanowires (SiNWs) exhibit a bandgap ranging from 1.5 to 1.8 eV, depending on their diameter [64], and similarly contribute to the formation of heterostructures when incorporated into composites. The MoS_2_-SiNWs adduct (Figure 6e) displayed a bandgap of 2.67 eV, aligning with the results observed in the catalysts (Figure 6c,d). This consistent reduction in bandgap is crucial as it enhances the composite’s ability to absorb visible light, increasing its applicability in photocatalytic processes.

The 5% (MoS_2_-SiNWs)@ZnONPs-CNTs catalyst showed a bandgap of 2.87 eV, placing it within the visible spectrum, and this shift was further shifted to 2.83 eV in the 6.7% (MoS_2_-SiNWs)@ZnONPs-CNTs catalyst. The continued incorporation of SiNWs, similar to MoS_2_, consistently reduced the bandgap, allowing for even greater visible light absorption and improved catalytic efficiency. This combination of materials not only shifts the optical absorption into the visible region but also facilitates improved charge separation and electron mobility, which are critical for photocatalytic applications. The results presented in Figure 6 clearly demonstrate that these catalysts are capable of efficiently utilizing visible light for catalytic processes, making them ideal candidates for applications such as hydrogen evolution reactions (HER), where the ability to harness a wide range of light wavelengths is paramount for enhancing overall catalytic performance. This conclusion is further reinforced by the hydrogen evolution results to be discussed later in the study.

#### 3.1.2. Photocatalytic Hydrogen Production

Before proceeding to the characterization of these catalysts in the hydrogen production reaction from water, an investigation into the optimal process conditions was conducted. For this investigation, the catalyst that exhibited the highest efficiency, namely 6.7% (MoS_2_-SiNWs)@ZnONPs-CNTs, was selected. The impact of the medium’s pH (Figure S1) and catalyst loading (Figure S2) was evaluated. The optimal conditions were found to be pH = 7 and a catalyst loading of 60 mg/100 mL. Control experiments (Figure S3) were also performed to determine whether the reaction could occur without light, thus distinguishing between purely catalytic and photocatalytic processes. As shown in Figure S3, the hydrogen production mechanism is predominantly photocatalytic, but in the absence of light, a small hydrogen yield of approximately 83 μmol/hg was observed, attributable solely to catalysis.

**Figure 7:**
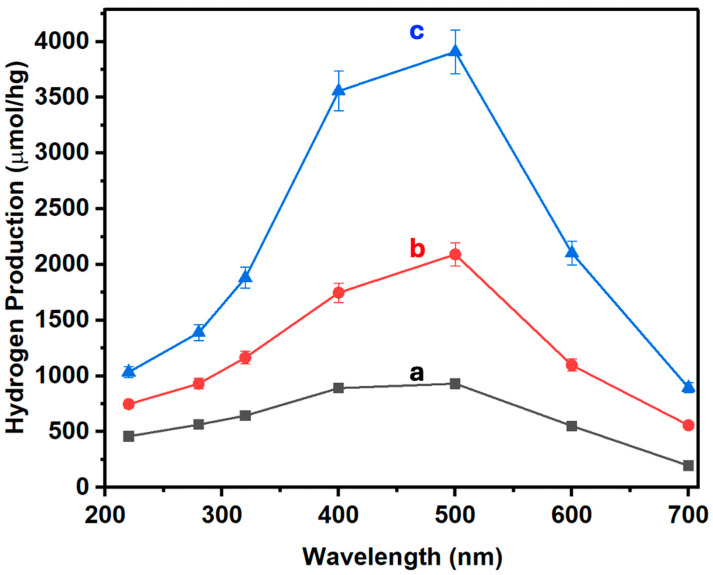
H_2_ production profiles of the synthesized materials under irradiation at different wavelengths. ZnONPs-CNTs (a); 5% (MoS_2_-SiNWs)@ZnONPs-CNTs (b); 6.7% (MoS_2_-SiNWs)@ZnONPs-CNTs (c). The vertical lines represent the average error of each measurement.

Figure 7 shows the results for hydrogen production via photocatalysis using the different materials studied. The photocatalytic efficiency was measured using Na_2_SO_3_ (0.02 M) and Na_2_S (0.4 M) as sacrificial agents. Activities were tested under different irradiation wavelengths of 220, 280, 320, 400, 500, 600, and 700 nm for all samples.

As illustrated in Figure 7, the base material (ZnONPs-CNTs) exhibits significant activity in the visible region, specifically between 400 and 500 nm (Figure 7a). When 5% of MoS_2_-SiNWs (comprising 3.3% MoS_2_ and 1.7% SiNWs) is added to this composite, there is a substantial increase in hydrogen production (Figure 7b). This enhancement is not limited to the visible region but extends across the entire spectral range studied. Moreover, when the percentage of MoS_2_ is increased to 5% (5% MoS_2_ and 1.7% SiNWs), the hydrogen production sees an even more pronounced boost, with the maximum observed production reaching 3909 μmol/hg at 500 nm. This significant enhancement underscores the synergistic effect of both additives (MoS_2_ and SiNWs) in enhancing the catalytic performance of the material. The results demonstrate that the integration of MoS_2_ and SiNWs into the CNTs-ZnONPs framework not only broadens the active spectral range but also dramatically amplifies the efficiency of hydrogen generation. The pronounced activity in the visible spectrum, coupled with the remarkable overall increase in hydrogen production, highlights the potential of these hybrid materials in photocatalytic applications. The synergy between MoS_2_ and SiNWs in the composite plays a vital role in enhancing charge separation and light absorption, thereby improving the overall photocatalytic performance. MoS_2_, with its layered structure and narrow bandgap (~1.8 eV), enhances light absorption, particularly in the visible spectrum, which allows the composite to capture a broader range of solar energy. The incorporation of SiNWs further enhances this effect by acting as a high-surface-area support, improving light scattering and interaction with the catalyst, which increases photon absorption. SiNWs also contribute to the formation of an extended heterojunction with MoS_2_, which facilitates more efficient charge separation by preventing recombination of photoexcited electron-hole pairs [63,64,65].

The formation of heterojunctions between MoS_2_, SiNWs, and CNTs-ZnONPs is crucial for efficient electron transfer. MoS_2_, in combination with SiNWs, creates a staggered energy band alignment, which promotes the transfer of photogenerated electrons from MoS_2_ to SiNWs, reducing the recombination rate. Additionally, the presence of CNTs provides conductive pathways for charge carriers, further enhancing the photocatalytic activity by promoting rapid electron transport [64]. This combination of enhanced light absorption and improved charge separation via heterojunction formation leads to the substantial increase in hydrogen production observed across the entire spectral range.

**Figure 8:**
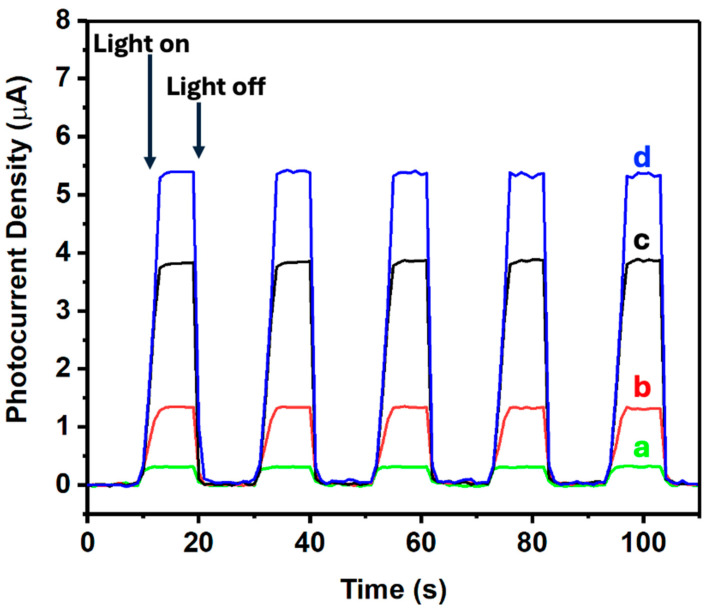
Transient photocurrent response in the light on–off processes of ZnONPs (a); ZnONPs-CNTs (b); 5% (MoS_2_-SiNWs)@ZnONPs-CNTs (c); and 6.7% (MoS_2_-SiNWs)@ZnONPs-CNTs (d), at 500 nm.

The photoelectrochemical properties of the different materials were investigated using a CHI660D electrochemical system in a 0.1 mol/L Na_2_SO_4_ solution (see Figure 8). Initially, 25 mg of the nanomaterial (either ZnONPs, CNTs-ZnONPs, 5% (MoS_2_-SiNWs)@ZnONPs-CNTs, or 6.7% (MoS_2_-SiNWs)@ZnONPs-CNTs) was dispersed in 3 mL of ethanol mixed with 10 μL of a 5 wt.% Nafion solution. Then, 200 μL of this suspension was applied to a 1 × 1 cm^2^ piece of fluorine-doped tin oxide (FTO) conductive glass, which served as the working electrode. The setup included a saturated calomel electrode (SCE) as the reference electrode and a 1 × 1 cm^2^ platinum sheet as the counter electrode. Transient photocurrent measurements were performed at a potential of 0.5 V (see Figure 8). Upon analysis, the transient photocurrent responses of the samples under cyclical light interruption at 500 nm revealed that ZnONPs exhibited a significantly lower photocurrent density compared to the CNTs-ZnONPs composite. However, when 5% (MoS_2_-SiNWs) were incorporated into this composite, a substantial enhancement in photocurrent was observed, with an increase up to almost 16 times the value shown by ZnONPs alone. This demonstrates that the addition of CNTs, and particularly the incorporation of MoS_2_-SiNWs, can effectively promote the dispersion of photo-induced carriers due to the formation of heterojunctions. This effect is even more pronounced when the MoS_2_ content is increased to 5% (5%MoS_2_-1.7%SiNWs), as seen in Figure 8d, where the hydrogen production under 500 nm radiation was analyzed. The results highlight the superior performance of the hybrid material, emphasizing its potential in enhancing photoelectrochemical processes. The creation of heterojunctions between the different components significantly improves charge separation and carrier mobility, thereby boosting the overall photocatalytic efficiency.

To further explore the impact of photogenerated carriers on hydrogen production, an additional study was performed using EDTA-Na_2_ as a hole (h^+^) scavenger. As shown in Figure S4, incorporating EDTA-Na_2_ into the reaction mixture led to a notable increase in hydrogen production for the most efficient catalyst, 6.7% (MoS_2_-SiNWs)@ZnONPs-CNTs, under irradiation across all tested wavelengths.

Previously presented results have already indicated that the catalysts in this study exhibit efficient electron–hole separation. However, the enhanced hydrogen production observed with the addition of EDTA-Na_2_ can be explained by the further reduction of electron–hole recombination, resulting in improved H_2_ generation. This additional scavenger study underscores the catalysts’ capability to maintain efficient charge separation and further optimizes their photocatalytic performance by mitigating recombination effects. The use of EDTA-Na_2_ provides a clearer understanding of the mechanisms at play and highlights the potential for further enhancing hydrogen production by fine-tuning the interaction between photogenerated carriers and scavengers.

A recyclability study of the most active catalyst, namely 6.7% (MoS_2_-SiNWs)@ZnONPs-CNTs, was conducted to assess its durability. The catalyst underwent 10 usage cycles. After each cycle, the catalyst was recovered from the reaction mixture via centrifugation at 5000 rpm for 15 min and subsequently washed through two centrifugation–washing cycles using water. The catalyst was then dried at 60 °C for 4 h in a vacuum oven before the next cycle. The results, as shown in Figure S5, indicate a noticeable decline in hydrogen production starting from the first cycle and continuing throughout the 10 cycles. After the final cycle, the measured H_2_ production was 3787 mol/hg, reflecting an efficiency drop of ca. 17%. This reduction in performance could be due to the potential leaching of MoS_2_-SiNWs during the catalyst’s use and regeneration processes. To further investigate this, a quantitative analysis of Mo and Si was performed using XPS. Initially, the elemental composition of the catalyst (6.7% (MoS_2_-SiNWs)@ZnONPs-CNTs) was: Mo 3.05%, S 1.98%, C 14.42%, Si 1.61%, Zn 61.33%, and O 17.64%, which provided a Mo/O ratio of 0.172 and a Mo/Si ratio of 1.89. After the 10th cycle of catalyst use, the composition changed, resulting in elemental ratios of Mo/O ratio of 0.113 and Mo/Si ratio of 1.30. This confirmed the possible leaching of Mo after the use and regeneration of the catalyst, which may explain the observed loss of efficiency over the cycles of catalyst use and regeneration. The remaining chemical elements did not exhibit significant changes that could be associated with leaching processes.

To elucidate the underlying mechanism, a comprehensive photoluminescence (PL) study was conducted on ZnONPs, ZnONPs-CNTs, and the catalysts 5% (MoS_2_-SiNWs)@ZnONPs-CNTs and 6.7% (MoS_2_-SiNWs)@ZnONPs-CNTs (Figure S6). The photoluminescence spectrum of ZnONPs alone (Figure S6a) exhibits a strong emission, indicative of significant electron–hole recombination within the material, which could limit its photocatalytic efficiency. Upon incorporating CNTs into the ZnONPs (Figure S6b), there is a slight decrease in luminescence intensity. This reduction suggests that the CNTs facilitate charge separation by providing efficient pathways for electron transfer, thereby reducing the likelihood of recombination. The catalyst 5% (MoS_2_-SiNWs)@ZnONPs-CNTs (Figure S6c) demonstrates a substantial reduction in fluorescence intensity, with the emission peak slightly red-shifted to approximately 547 nm. This red shift, coupled with the decreased PL intensity, aligns with the hypothesis that the introduction of MoS_2_ and SiNWs into the heterostructure effectively suppresses electron–hole recombination. The red shift may also indicate enhanced electron transfer processes, as the formation of more effective catalytic sites within the heterostructure leads to improved photocatalytic activity. In the 6.7% (MoS_2_-SiNWs)@ZnONPs-CNTs catalyst (Figure S6d), where the MoS_2_ content is further increased, the fluorescence intensity decreases even further. The emission maximum remains nearly identical to that observed in Figure S6c, suggesting that the additional MoS_2_ continues to enhance charge separation efficiency, but the system may have reached a saturation point where further increases in MoS_2_ content do not significantly alter the emission properties. The findings from this photoluminescence study establish a clear correlation between the incorporation of various cocatalysts into the ZnONPs structure and the substantial reduction in electron–hole recombination processes. The enhanced charge separation efficiency, as evidenced by the decreased PL intensity and red shift, indicates that these catalysts are particularly effective at minimizing recombination losses. This, in turn, maximizes the availability of charge carriers for the hydrogen evolution reaction (HER), ultimately leading to superior catalytic performance.

**Figure 9:**
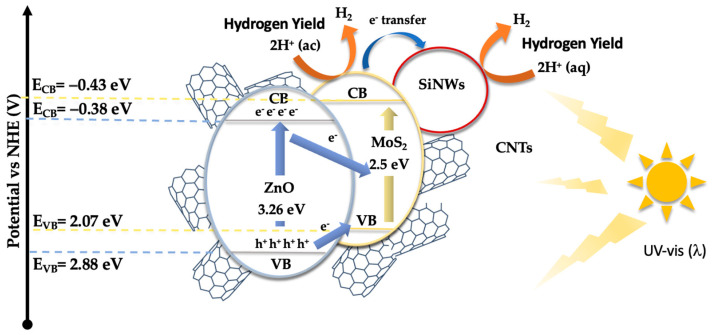
Schematic diagram of the proposed mechanism for hydrogen production under UV–visible light irradiation.

Based on these results and the established bandgaps (see Figure 6), a catalytic mechanism has been proposed (Figure 9). The Mulliken electronegativity concept [65,66] was applied to determine the band edge positions of the catalyst’s components, which allowed for the identification of the pathways followed by the photogenerated charge carriers within the composite material (see Equations (1) and (2)).

E_CB_ = X − E_C_ − 0.5E_g_ (1)

E_VB_ = E_CB_ + E_g_ (2)

In these equations, E_CB_ and E_VB_ represent the conduction band (CB) and valence band (VB) edge potentials, respectively. X is the absolute electronegativity, and E_C_ is the free electron energy on the hydrogen scale (4.50 eV) [67]. The values of X for ZnONPs, MoS_2_, and SiNWs are 5.75 eV [68], 5.32 eV [69], and 4.3 eV [70], respectively. With a bandgap (Eg) of 3.26 eV for ZnONPs, the calculated conduction and valence band edge positions for ZnONPs are −0.38 eV and 2.88 eV, respectively. For MoS_2_, the corresponding band positions are −0.43 eV for the CB, and 2.07 eV for the VB. For SiNWs, the computed band edge positions are −0.98 eV, and 0.58 eV, respectively. These calculations provide critical insights into the relative alignment of the energy bands, which is essential for understanding the flow of photogenerated electrons and holes within the composite.

Figure 9 presents a schematic representation of the proposed catalytic mechanism for the hydrogen evolution reaction (HER). In this mechanism, ZnONPs and MoS_2_ serve as the primary light-absorbing materials, with bandgaps of 3.26 eV for ZnONPs and approximately 2.5 eV for MoS_2_. When exposed to radiation, ZnONPs absorb photons, resulting in the excitation of electrons from the valence band (VB) to the conduction band (CB). The conduction band edge of ZnO is positioned at −0.38 eV, while its valence band edge is at 2.88 eV relative to the normal hydrogen electrode (NHE). The photogenerated electrons in the conduction band of ZnONPs are then transferred, possibly through a Z-scheme mechanism, to MoS_2_. This alignment facilitates the efficient transfer of electrons from ZnO to MoS_2_, enabling electron promotion across a broad range of wavelengths. Simultaneously, silicon nanowires (SiNWs), which may act as electron sinks as described in other studies, facilitate reduction processes leading to H_2_ production. MoS_2_ also serves as an active site where the reduction of protons (H^+^) occurs, ultimately producing hydrogen gas (H_2_). This mechanism explains the effect of MoS_2_ and SiNWs incorporation on the reduction of electron–hole recombination, as evidenced by photoluminescence studies. Additionally, carbon nanotubes play a critical role in this system by providing a conductive network that enhances electron transport from ZnONPs to MoS_2_ and SiNWs. This network reduces electron–hole recombination, thereby improving the efficiency of charge separation. The integration of CNTs within the catalyst not only maintains structural integrity but also ensures that electrons are efficiently delivered to the active sites. As the photogenerated electrons are transferred through the composite, they participate in proton reduction to generate hydrogen. Meanwhile, the holes left in the valence band of ZnONPs may also engage in oxidation reactions, although the primary focus here is on the reduction processes that lead to hydrogen evolution.

As summary, the catalytic efficiency of the MoS_2_-SiNWs-SWNTs@ZnONPs composites in hydrogen production, observed under 500 nm wavelength irradiation and yielding ca. 3909 µmol h^−1^ g^−1^, is notably high when compared with published results. This efficiency significantly surpasses that of simpler catalytic systems, such as MoS_2_-TiO_2_ nanocomposites, which achieved a maximum of 150.7 µmol h^−1^ g^−1^ for a composition containing 4.0 wt.% MoS_2_-TiO_2_ [71]. Furthermore, even advanced MoS_2_/g-C_3_N_4_ composites with an optimized flower-like MoS_2_ structure reached only up to 867.6 µmol h^−1^ g^−1^ [72]. These results underscore the superior hydrogen production capability of the MoS_2_-SiNWs-SWNTs@ZnONPs composites, attributed to the synergistic interaction among silicon nanowires (SiNWs), single-walled carbon nanotubes (SWNTs), and MoS_2_ within a zinc oxide nanoparticle (ZnONP) matrix. The strategic integration of these components enhances electron transport and improves light absorption, resulting in significantly elevated photocatalytic activity under visible light. Nevertheless, despite the excellent performance of the MoS_2_-SiNWs-SWNTs@ZnONPs composites, more complex ternary MoS_2_-based systems can exhibit even higher catalytic efficiencies. For instance, MoS_2_/ZnCdS/ZnS nanocomposites have demonstrated hydrogen production rates reaching up to 79.3 mmol g^−1^ h^−1^ [73], highlighting the potential for further enhancements in MoS_2_-based photocatalytic systems through the inclusion of additional semiconductors and co-catalysts. These systems often benefit from multiple mechanisms of synergy between their components, such as improved charge carrier separation, a broadened light absorption spectrum, and efficient spatial separation of oxidation and reduction sites, all of which collectively contribute to their high performance. Our results suggest potential pathways for further optimizing the MoS_2_-SiNWs-SWNTs@ZnONPs composite. Future work, which is already underway, aims to explore the integration of additional semiconductor materials that could introduce new charge separation mechanisms or further extend the light absorption range. Additionally, fine-tuning the proportions and distribution of MoS_2_, SiNWs, and SWNTs within the composite could optimize the interface interactions and electronic properties, potentially leading to even higher rates of hydrogen production.

### 3.2. Li-Ion Batteries

#### 3.2.1. Morphology of Electrodes

**Figure 10:**
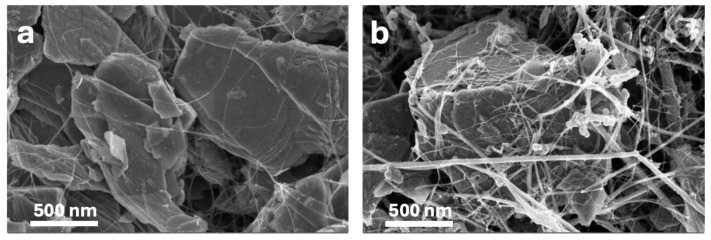
FESEM images of MoS_2_@CNTs (**a**), and MoS_2_@SiNWs@CNTs (**b**) composites.

**Figure 11:**
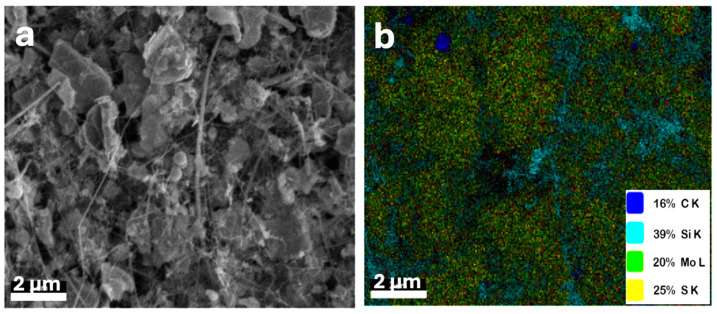
FESEM image of the MoS_2_@SiNWs@CNTs composite (**a**), and elemental EDS mapping (**b**).

The morphology of the synthesized electrodes, including pure MoS_2_, MoS_2_@CNTs (80:20 wt.%), and MoS_2_@SiNW@CNTs (56:24:20 wt.%) composites, was investigated by FESEM. The pure MoS_2_ electrode consists of irregularly shaped flakes, predominantly measuring between 1 to 1.5 μm. FESEM images distinctly reveal the layered structure of these sheets, while transmission electron microscopy (TEM) images confirm their nanoscale thickness, indicating the presence of nanometric steps. Upon the introduction of CNTs to the MoS_2_ nanosheets (see Figure 10a), a thin network of nanotubes is observed to permeate through the MoS_2_ layers, facilitating interconnection. The incorporation of SiNWs alongside CNTs does not markedly alter the overall morphology (Figure 10b); instead, it forms a denser network comprising SiNWs and CNTs, which envelops the MoS_2_ layers, leading to the formation of a more uniform composite structure, as confirmed by energy dispersive X-ray spectroscopy (EDX) analysis (Figure 11).

The integration of CNTs and SiNWs into MoS_2_ is anticipated to significantly enhance the electrochemical performance of the resultant electrodes. Pure MoS_2_ exhibits a tendency to aggregate, which compromises its performance during cycling. The inclusion of CNTs enhances the electrical conductivity, thereby improving electron transport within the electrode and enabling faster charge/discharge rates. This improved conductivity is expected to result in superior electrochemical performance, including increased specific capacities and enhanced rate capabilities. Moreover, the incorporation of SiNWs within the MoS_2_ matrix aids in mitigating aggregation during charge and discharge cycles, particularly under high current densities. This is achieved by providing structural stability, which prevents issues such as layer exfoliation and material degradation. Consequently, the combined presence of CNTs and SiNWs not only reduces volume expansion and aggregation but also enhances the capacity and stability of the electrode material. These findings suggest that the incorporation of CNTs and SiNWs contributes to improved stability and longevity of the composite under high energy density conditions. Additionally, these composites offer the advantage of tunability, allowing for optimization of conductivity, capacity, and mechanical strength to meet specific application requirements. Overall, MoS_2_-based composites reinforced with CNTs and SiNWs are promising candidates for high-performance applications in energy storage and conversion technologies [74,75,76].

#### 3.2.2. Electrochemical Study

**Figure 12:**
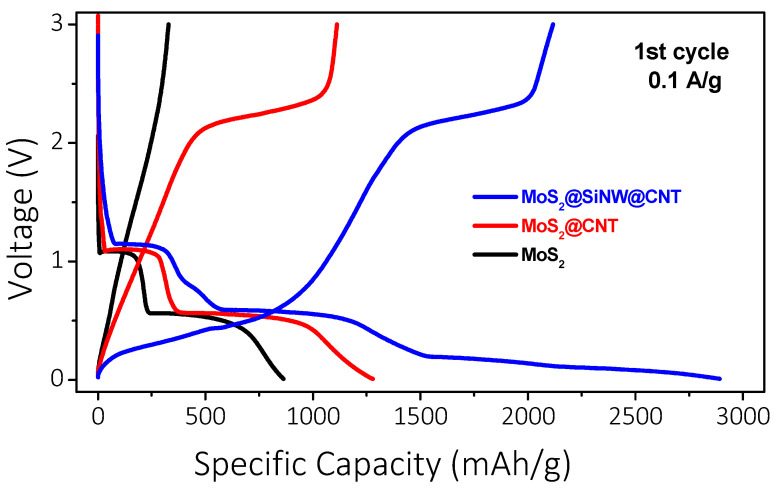
GCD curves for MoS_2_-based electrodes, showing no reversibility for pure MoS_2_ and higher capacity retention for MoS_2_@SiNW@CNTs compared to MoS_2_@CNTs.

The electrochemical performance of MoS_2_-based electrodes as an anode material for Li-ion batteries was investigated through galvanostatic charge/discharge (GCD) cycling. Initially, exfoliated pure MoS_2_ was tested by cycling coin cells in a half-cell configuration. In the first discharge curve (Figure 12), recorded at a current density of 0.1 A/g, two distinct plateaus were observed at 1.09 V and 0.56 V, corresponding to the initial insertion of lithium ions with the formation of Li_x_MoS_2_ and the subsequent formation of lithium sulfide (Li_2_S) along with the solid electrolyte interphase (SEI) layer, respectively [77,78,79]. A specific capacity of 856 mAh/g was achieved; however, no further transitions were detected during the charge cycle, indicating the absence of a reversible process. These results show the irreversible behavior of pure MoS_2_, likely due to the agglomeration of MoS_2_ sheets, the formation of a non-conductive SEI, and insufficient electrical conductivity. Additionally, the SEI may have exhibited high electrical resistance and instability, further contributing to the rapid capacity fade observed. To address the issues observed with pure MoS_2_, a composite of MoS_2_ and carbon nanotubes (MoS_2_@CNT, 80:20 wt.%) was tested in new cells. The introduction of CNTs with the MoS_2_ active material enabled the reversibility of the electrodes, as shown in Figure 12. The first discharge curve exhibited longer plateaus at 1.14 V and 0.56 V, with charging transitions around 2.2 V, indicating clear redox processes and high reversibility.

**Figure 13:**
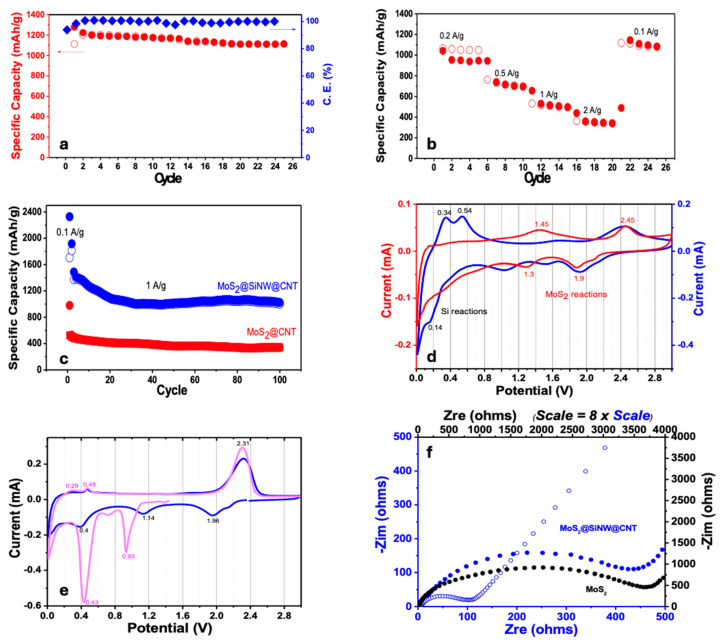
Electrochemical study. Galvanostatic charge–discharge (GCD) curves of the MoS_2_@CNTs composite electrode at a current density of 0.1 A/g for 25 cycles, showing high specific capacity and good cycling stability (**a**). Rate performance of the MoS_2_@CNTs composite electrode at varying current densities from 0.2 to 2.0 A/g, demonstrating excellent capacity retention when returned to 0.1 A/g (**b**). Extended cycling performance of the MoS_2_@CNT and MoS_2_@SiNW@CNT composite electrodes at 1 A/g over 100 cycles, with the MoS_2_@SiNW@CNT composite achieving higher specific capacity and enhanced stability, maintaining a capacity of ~1000 mAh/g after 100 cycles (**c**). Cyclic voltammograms (CV) after 100 cycles, showing distinct redox peaks for MoS_2_ and SiNWs in the MoS_2_@SiNW@CNT composite electrode (**d**). CVs for the first and second cycle of MoS_2_@SiNW@CNT electrode (**e**). Electrochemical impedance spectroscopy (EIS) plots of MoS_2_@CNT and MoS_2_@SiNW@CNT electrodes, showing lower charge transfer resistance in the SiNW-containing composite, contributing to its superior electrochemical performance (**f**).

Figure 13a illustrates the enhanced electrochemical performance obtained in a MoS_2_@CNT composite electrode, which demonstrated superior stability over 25 cycles at a current density of 0.1 A/g. The composite electrode achieved a high specific capacity of 1278 mAh/g during the first discharge, with a corresponding charge capacity of 1112 mAh/g, resulting in a coulombic efficiency of 93.83%. Moreover, the electrode showed excellent cycling performance, maintaining a stable specific capacity around 1100 mAh/g, with a coulombic efficiency of 99.67%, after 25 cycles. Following this, the rate performance of the MoS_2_@CNT composite electrode was evaluated at various current densities (Figure 13b), including 0.2, 0.5, 1.0, and 2.0 A/g, before returning to 0.1 A/g. At these higher current densities, the electrode exhibited reversible capacities of 1000, 700, 500, and 350 mAh/g, respectively. Notably, upon reverting to the lower current density of 0.1 A/g, the electrode demonstrated a remarkable recovery of its capacity to 1080 mAh/g, which is close to its initial value. This result highlights the excellent rate capability and cycling stability of the MoS_2_@CNT composite, indicating its robust performance and ability to maintain high capacity even under varying charge and discharge conditions. All these significant performance enhancements can be attributed to the increased electrical conductivity and structural stability provided by the CNTs [80], which support and stabilize the MoS_2_ sheets and facilitate the reversibility of redox processes in the battery. Following the favorable electrochemical performance observed in the MoS_2_@CNTs composite, extended cycling tests were conducted at a higher current density of 1 A/g. Figure 13c illustrates the GCD curves of these composites in red symbols. The first two cycles were performed at a low current density of 0.1 A/g to ensure the formation of a stable SEI layer on the electrodes. The remaining cycles, up to 100, were measured at the higher current density of 1 A/g. The GCD curves of the MoS_2_@CNTs composite reveal the strong reduction of the specific capacity (from 970 to 520 mAh/g) from 0.1 A/g to 1 A/g, and a significant decline over cycling, with the capacity decreasing from 500 to 400 mAh/g during the first 25 cycles and reaching around 350 mAh/g after 100 cycles. To develop electrodes with enhanced capacity and shorter cycling times, alternative composites were proposed. New composites incorporating silicon nanowires (SiNWs) were investigated, with composition MoS_2_@SiNW@CNT (56:24:20 wt.%). These new composites showed significant improvements in both stability and capacity compared to the previous materials. Figure 13c also illustrates the electrochemical performance of the MoS_2_@SiNW@CNT composite (blue symbols) under the same cycling conditions as the MoS_2_@CNT electrode. A significant increase in specific discharge capacity is observed, reaching 2330 mAh/g in the first cycle and 1915 mAh/g in the second cycle at 0.1 A/g, despite an irreversible loss of capacity. The composite demonstrates good performance at high current density, with a gradual decrease in capacity from 1400 to 1000 mAh/g during the first 30 cycles at 1 A/g, followed by excellent cyclic stability over extended cycling periods, maintaining a capacity of 990 mAh/g up to the 100th cycle.

When dealing with two active materials like Si and MoS_2_ in a composite electrode, it is crucial to accurately calculate the overall specific capacity. This is influenced by the individual capacities of Si and MoS_2_ and their respective weight fractions in the composite. Si offers a much higher theoretical specific capacity (4200 mAh/g) compared to MoS_2_ (669 mAh/g) [81,82]. Therefore, determining the ratio of Si to MoS_2_ is essential for predicting and optimizing the composite’s electrochemical performance. Taking into account the proportion of each active material, the MoS_2_@SiNW@CNT (56:24:20 wt.%) composite cycled at 1 A/g corresponds to 0.578 C. This C-rate is equivalent to 1.5 C for the MoS_2_@CNT (80:20 wt.%) composite, which cycles at a current density of 386.5 mA/g (~0.4 A/g). A realistic comparison in specific capacities, under the same C-rate and cycling conditions, shows that introducing SiNWs into MoS_2_@CNT, changing the active material composition from 100% MoS_2_ to 70% MoS_2_ and 30% SiNWs, leads to a capacity increase from 800 mAh/g for MoS_2_@CNT (see Figure 13b for rates between 0.2 A/g and 0.5 A/g) to 1000 mAh/g for MoS_2_@SiNW@CNT (see Figure 13c at 1 A/g). The incorporation of SiNWs and MoS_2_ into the composite, combined with CNTs, significantly enhances the electrochemical performance by creating a more robust electrode structure. This synergy between MoS_2_, SiNWs, and CNTs results in a 20% increase in specific capacity (at equivalent C-rates) and, most importantly, enables long cycling at high current densities, effectively overcoming the limitations observed in MoS_2_@CNT composites. The electrochemical performances of both MoS_2_@CNT and MoS_2_@SiNW@CNT composites were investigated by cyclic voltammetry (CV). Representative CV of composites after long cycling at high current density in the voltage range of 0.01–3.0 V at a scan rate of 0.2 mV/s are shown in Figure 13d. Distinct peaks are observed, corresponding to the reactions of the active materials: layered MoS_2_ (indicated by red voltage values) and Si nanowires (indicated by black voltage values), as explained below. For the MoS_2_@SiNW@CNT electrode, voltammetry of the first and second cycles was also recorded, as shown in Figure 13e. During the initial discharge (pink curve, Figure 13e), the reduction process (cathodic sweep) in the MoS_2_@SiNW@CNT composite primarily exhibits behavior characteristic of reactions of MoS_2_ with Li, as indicated by the presence of two distinct reduction peaks—specifically, a minor reduction peak at 0.93 V and a more pronounced reduction peak at 0.43 V, suggesting a complex multi-step reduction process. The 0.93 V peak is likely due to the transition of MoS_2_ from a trigonal prismatic to an octahedral structure as lithium ions intercalate, consistent with previous reports [83]; it is a two-phase transition and, consequently, the peak is narrow. The 0.43 V reduction peak is attributed to the conversion of MoS_x_ into Mo nanoparticles within a Li_2_S matrix process [84] and the formation of the SEI layer. The peak at 0.43 V does not appear at subsequent cycles (see Figure 13d). This absence indicates these reactions are predominantly irreversible. After the first cycle, the electrode’s structure, including MoS_2_ and SiNWs, undergoes significant changes, leading to the formation of a stable SEI layer and altering the electrochemical response in later cycles. Also, it should be noted that this specific CV curve (pink curve, Figure 13e) starts at 1.4 V, so any additional step-down peaks occurring at higher voltages may be present but are not captured in the plot. On the oxidation curve (anodic sweep) of the first cycle, two small peaks at low voltages, 0.29 V and 0.48 V, indicate the oxidation processes of Li and Si. The peak at 0.29 V is likely related to the oxidation of lithium (Li) where lithium ions (Li^+^) are released into the electrolyte, possibly forming lithium compounds such as Li_2_O or Li_2_CO_3_ on the electrode surface. The peak at 0.48 V corresponds to the oxidation of silicon, where Si reacts with Li^+^ to form lithium silicides (Li_4_Si or similar compounds) or other lithium-silicon intermetallic [85]. Additionally, a prominent peak at 2.31 V is observed, associated with the re-oxidation of molybdenum and lithium sulfide back to MoS_2_, indicating the regeneration of the MoS_2_ structure from Mo and Li_2_S.

In the second discharge–charge cycle (blue curve, Figure 13e) of MoS_2_@SiNW@CNT, a new reduction peak appears at 1.96 V that was not recorded in the first cycle, as indicated above. This peak may correspond to the formation of new lithium intercalation compounds or phases that develop as the electrode material evolves during cycling. The narrow peaks observed in the first cycle at 0.93 V and 0.43 V become rounded, decrease in intensity, and shift slightly to 1.14 V and 0.40 V, respectively. Such changes are commonly attributed to the alteration of the electrode’s microstructure and the formation of SEI layers that affect peak characteristics over multiple cycles. For the oxidation curve, only a small decrease in the intensity of the peak at 2.3 V is observed, reflecting the reversible behavior of the electrode and indicating that the re-oxidation process of MoS_2_ is largely preserved despite cycling.

Each representative peak observed in the CV curves of the MoS_2_@SiNW@CNT composite corresponds to the distinct intercalation and conversion reactions of MoS_2_ and Si during discharge and charge, as detailed below (refer to Figure 13d for a visual representation).

**During discharge (intercalation at the working electrode)** [86,87]:
(1) *x*Li^+^ + *x*e^−^ *+* MoS_2 →_ Li_x_MoS_2_
(1b) Li_x_MoS_2_ + (4 − *x*) Li^+^ + (4 − *x*)e^−^ _→_ Mo + 2Li_2_S
(2) *x*Li^+^ + *x*e^−^ *+* Si (crystalline) _→_ Li_x_Si (amorphous)
(3) Li_x_Si (amorphous) + (3.75 − *x*)Li^+^ *+* (3.75 − *x*)e^−^ _→_ Li_15_Si_4_ (crystalline)
**During charge (conversion reaction at the working electrode)** [87,88,89]:
(4) Li_15_Si_4_ (crystalline) _→_ 4Si (amorphous) + 15Li^+^ *+* 15e^−^
(5) Li_2_MoS_2 →_ MoS_2_ + 2Li^+^ *+* 2e^−^
(6) Mo + 2Li_2_S _→_ MoS_2_ + 4Li^+^ *+* 4e^−^
**Reduction (Cathodic sweep)**
(1) Reduction Peak at 1.9 V: This peak corresponds to the initial insertion of lithium ions into the MoS_2_ structure. During this stage, MoS_2_ undergoes a phase transformation, where Li ions intercalate into the MoS_2_ layers, leading to the formation of Li_x_MoS_2_. This process is reversible and is characteristic of the layered structure of MoS_2_.
(1b) Reduction Peak at 1.3 V: This peak is associated with the further reduction of MoS_2_ to form lithium sulfide (Li_2_S) and metallic molybdenum (Mo).
(2,3) Small reduction peak at 0.14 V: The reactions of Si and Li occur at low voltages [90] and may not be clearly detected in CV reduction curves recorded at 0.2 mV/s. The lithiation of silicon nanowires follows reaction (2) and is observed only in the first cycle. Subsequently, more Li is incorporated into the amorphous alloy Li_x_Si, following reaction (3). These transformations are evident in the oxidation curves, where corresponding peaks are observed, and are clearly seen in the dQ/dV derivative curves from GCD (see Figure S7). These dQ/dV curves are essential for detecting redox transitions in electrochemical systems, revealing subtle voltage changes linked to oxidation and reduction processes and providing insights into the material’s redox behavior and stability during cycling. Therefore, the small peak at 0.14 V is attributed to reaction (3), representing the lithiation of silicon nanowires.
**Oxidation (Anodic sweep)**
(4) Oxidation peaks at low voltages (0.34 and 0.52 V): These peaks indicate the initial delithiation of lithium silicide (Li_x_Si) and are observed exclusively in the MoS_2_@SiNW@CNT composite (blue curve in Figure 13d). The peak at 0.34 V is likely associated with the delithiation of LiSi, where lithium is extracted from the silicon component, leading to the formation of silicon and the corresponding release of lithium ions (reaction 4). Similarly, the peak at 0.52 V may reflect the delithiation of intermediate phases or lithium silicide compounds, suggesting that multiple stages of lithiation and delithiation occur. These processes are characteristic of the interactions between lithium and silicon nanowires within the composite, highlighting the active role of Si in the electrochemical behavior of the MoS_2_@SiNW@CNT composite. The observation of these peaks only in the composite indicates that the presence of SiNWs significantly impacts the electrochemical response, distinguishing it from other materials like MoS_2_ alone.
(5, 6) Oxidation peaks at higher voltages (1.45 and 2.45 V): The peak at 1.45 V corresponds to the initial delithiation of the Li_x_MoS_2_ phase, where lithium ions are extracted (reaction 5), reversing the intercalation process. This process may not completely revert to the original MoS_2_ structure, leading to partial reformation of the MoS_2_ phase. The peak at 2.45 V is associated with the re-oxidation of molybdenum and lithium sulfide back to MoS_2_ (reaction 6), indicating the regeneration of the MoS_2_ structure from Mo and Li_2_S. This peak represents the final oxidation step, completing the delithiation process and restoring the electrode to its original state.

As previously mentioned, the reactions of Si and Li are well defined in the differential capacity (dQ/dV) curves derived from GCD profiles. Figure S7b shows the dQ/dV curves for MoS_2_@SiNW@CNT at a current density of 0.1 A/g. Reduction/oxidation transitions are observed around 0.3/0.4 V and 0.2/0.3 V, respectively, which are attributed to the electrochemical redox reactions of Si. These observations are consistent with the results obtained using pure SiNW electrodes (without MoS_2_ and CNT) [91]. Accordingly, the peaks at low voltages are absent in MoS_2_@CNT (Figure S7a, 0.1 A/g MoS_2_@CNT). The transitions of MoS_2_ occur at higher voltages and are present in both composites’ electrodes, with redox transitions around 1.9/2.3 V and 1.1/1.7 V. It is worth noting that as cycling progresses, the main peaks for redox (1.9/2.3 V) undergo more significant changes in decreasing/increasing intensities and shift to lower/higher voltages in the MoS_2_@CNT electrodes compared to the MoS_2_@SiNW@CNT electrodes, indicating that the SiNW structure helps stabilize the redox behavior, potentially leading to enhanced cycling stability and performance of the composite material.

Finally, it is important to note that the transitions are also observed when cycling at a high current density (1 A/g) (Figure S7c). However, the double peaks at low voltages merge into a single, more rounded peak. The good recyclability and stability of the MoS_2_@SiNW@CNT composite are still evident, as indicated by the minimal variation in voltages and slight changes in the intensities of the peaks corresponding to the redox transitions of MoS_2_ up to the 50th cycle.

The electrochemical impedance spectroscopy (EIS) plots of pure MoS_2_ and the MoS_2_@SiNW@CNT composite before cycling are shown in Figure 13f. It is evident that the conductivity of MoS_2_ is much lower than that of MoS_2_@SiNW@CNT, as indicated by the two different X scales of the figure, which reveal a charge transfer resistance more than seven times higher for MoS_2_ compared to MoS_2_@SiNW@CNT. During the electrochemical reaction, the SiNWs and CNTs enhance the conductivity of MoS_2_ and reduce the charge transfer resistance, potentially leading to faster Li-ion diffusion and improved electrochemical performance, as discussed earlier. After the first CV cycle, EIS measurements were also recorded for the MoS_2_@SiNW@CNT electrode, showing an impressive reduction in resistance by almost five times. This significant decrease in resistance underscores the effectiveness of the SiNW and CNT components in enhancing the overall conductivity and performance of the composite material. This innovative composite integrates MoS_2_ and CNTs with SiNWs. The synergy between these materials leverages their individual advantages: MoS_2_ serves as the main active material, providing significant electrochemical activity and capacity; CNTs enhance structural stability and conductivity, as previously corroborated; and SiNWs contribute additional capacity, electrochemical activity, and stability at high current densities. The performance of these new composites demonstrated significant improvements over the previous materials. Specifically, they exhibited enhanced stability during extended cycling and a notable increase in capacity. The improved performance attributes make MoS_2_@SiNW@CNTs a promising candidate for advanced electrode materials in high-capacity and high-performance energy storage applications.

### 3.3. Postmortem Study

**Figure 14:**
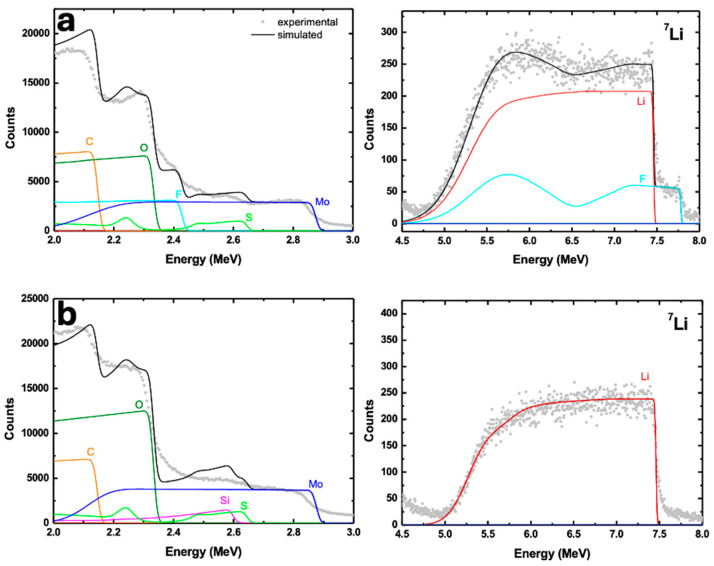
Postmortem analysis of the cycled MoS_2_@CNT (80:20 wt.%) (**a**) and MoS_2_@SiNW@CNT (56:24:20 wt.%) (**b**) electrodes after 100 cycles at 1 A/g using nuclear reaction analysis (NRA). The measurements were performed at a scattering angle of 170°. The spectra show the concentration depth profiles of Li and other elements (C, O, S, Mo) in the electrodes, revealing significant Li penetration in the MoS_2_@SiNW@CNT electrode, indicating improved Li diffusion and retention (right spectra); and corresponding Rutherford backscattering spectrometry (RBS) spectrum highlighting the distribution of heavier elements in the post-cycled electrodes (left spectra), confirming the presence of a stable solid electrolyte interphase (SEI) layer and the structural integrity of the MoS_2_@SiNW@CNT composite, which contributes to the long-term electrochemical stability observed during cycling.

A postmortem study of the electrodes was conducted using SEM imaging and ion beam analysis techniques as nuclear reaction analysis (NRA measurements). Figure S8 presents cross-sectional SEM images of pristine MoS_2_@CNT (80:20 wt.%) (Figure S8a) and MoS_2_@SiNW@CNT (56:24:20 wt.%) (Figure S8c) composites. After subjecting the cells to 100 charge–discharge cycles at a high current density of 1 A/g, the batteries were disassembled as outlined in the experimental section, and the cycled electrodes were analyzed (Figure S8b and S8d, respectively). SEM analysis of the cycled electrodes reveals a marked increase in electrode thickness compared to the pristine samples, indicating significant volumetric expansion. This substantial thickening is likely due to the formation of a thick solid-electrolyte interphase (SEI) layer. Figure 14 shows the spectra of the postmortem electrodes, obtained using 3-MeV H^+^. The total spectrum has been divided into two graphs: one highlighting the Li signal obtained through nuclear reactions (i.e., the NRA spectrum at higher energies), and another showing the region below 3 MeV, which represents the Rutherford backscattering spectrometry (RBS) processes where the other elements are observed. This setup allows for the determination of the concentration depth profile of Li atoms. A quantitative analysis of the Li depth profiles was performed using SIMNRA simulations, with both experimental and simulated data plotted in the corresponding graphs. For the MoS_2_@CNTs (80:20 wt.%) composite (Figure 14a), the SIMNRA simulations yielded the following atomic concentrations: 22.5% Li, 24.3% C, 21.1% O, 25.3% F, 4.5% S, and 2.23% Mo. These concentrations reflect mass increases of 35.08% from Li, 45.18% from excess C, 75.13% from O, and 107.17% from F. For the MoS_2_@SiNW@CNTs (56:24:20 wt.%) composite (Figure 14b), the NRA spectra results indicate a slightly higher concentration of Li after cycling. The presence of Si is less clear, potentially due to dilution effects, and no detectable signals for F or Fe are detected. However, there is a noticeable increase in carbon concentration beyond the expected value, as well as a significant amount of oxygen. The atomic concentrations obtained are 28.9% Li, 21.7% C, 35.0% O, 0% F, 5.8% Si, 5.8% S, and 2.9% Mo. The corresponding mass increases relative to the original composition are 27.5% due to Li, 8.4% due to increased C, and 76.7% due to O (Table S2). For both electrodes, it is observed that Li has penetrated significantly into the bulk volume, according with the good performance exhibited for the corresponding batteries. The introduction of SiNWs into the composite seems to further enhance this penetration, suggesting an improvement in the electrochemical behavior. The concentrations of Mo and S align well with those expected for MoS_2_, confirming the integrity of the MoS_2_ structure post-cycling. However, the excess carbon detected could be due to carbonaceous byproducts formed during cycling, such as those from the solid electrolyte interphase (SEI) or electrolyte decomposition. Additionally, the cleaning process with DMT may have increased surface reactivity, leading to further carbon adsorption, especially when exposed to the atmosphere. This combination of intrinsic material reactivity and post-cleaning atmospheric contamination likely contributes to the observed excess carbon, though it is less likely to explain the uniform distribution across the entire electrode volume. This increase in carbon, while unexpected, does not appear to adversely affect the battery performance, which remains strong. These results highlight the effectiveness of the prepared electrodes, particularly the MoS_2_@SiNW@CNTs composite, in maintaining structural integrity and facilitating efficient Li diffusion, making them promising candidates for high-performance lithium-ion batteries.

## 4. Conclusions

The study successfully demonstrated the synthesis and performance evaluation of MoS_2_-SiNWs-SWNTs@ZnONPs nanocomposites for HER and MoS_2_-SiNWs-SWNTs composites for Li-ion battery applications. For HER, the integration of MoS_2_, SiNWs, and SWNTs into a ZnO nanoparticle (ZnONP) matrix resulted in a significant enhancement in catalytic efficiency under visible light irradiation. The optimized composite, containing 6.7% MoS_2_-SiNWs, achieved an impressive hydrogen production rate of approximately 3909 µmol h^−1^ g^−1^ under 500 nm light, more than 40 times higher than pristine ZnONPs. This improvement is due to the synergistic effects of the materials, which enhance charge separation, extend the absorption spectrum into the visible range, and provide more active sites for the HER. MoS_2_ played a key role in enhancing the photocatalytic efficiency, while the combination with SWNTs and SiNWs further improved electron transport and stability, making the composite highly effective for practical hydrogen production.

The proportions of individual components in high-performance composites, such as those for photocatalytic hydrogen production, are carefully optimized to achieve a balance between light absorption, charge separation, and surface area. In the 6.7% (MoS_2_-SiNWs)@ZnONPs-SWNTs composite, this specific percentage of MoS_2_ was found to be optimal for extending visible light absorption while facilitating efficient charge separation due to the formation of heterojunctions between MoS_2_, SiNWs, and SWNTs. Increasing the MoS_2_ content beyond this level may lead to aggregation of MoS_2_ nanosheets, reducing the available surface area and limiting further improvements in photocatalytic performance. Additionally, while MoS_2_ enhances the material’s light-harvesting capabilities, excessive amounts can negatively impact both the mechanical stability and catalytic efficiency of the composite. Therefore, further increasing the MoS_2_ content may not necessarily improve photocatalytic performance as it could hinder the delicate balance between these properties.

In contrast, for lithium-ion batteries, the study focused on the MoS_2_-SiNWs-SWNTs composites without ZnONPs as electrode materials. These composites exhibited excellent electrochemical performance, particularly in terms of specific capacity and cycling stability. The MoS_2_@SiNWs@SWNTs composite (56:24:20 wt.%) demonstrated a high specific capacity of up to 1000 mAh g^−1^ after 100 cycles at a current density of 1 A g^−1^. The integration of SiNWs provided mechanical stability, mitigating issues such as volume expansion and aggregation that typically affect the cycling performance of silicon-based electrodes. Meanwhile, the inclusion of SWNTs enhanced electrical conductivity, improving electron transport during charge–discharge cycles and contributing to higher capacity retention.

Despite these promising results, challenges remain, particularly in the durability of the HER catalysts, where a decline in hydrogen production was observed after several cycles. This degradation is likely due to the leaching of MoS_2_ during operation and regeneration. Future work should focus on improving the stability of the composites, possibly through the addition of stabilizing agents or surface modifications to prevent material loss. Additionally, exploring alternative co-catalysts or semiconductor materials may further enhance both the photocatalytic and electrochemical performance of the composites. In conclusion, the MoS_2_-SiNWs-SWNTs@ZnONPs nanocomposites for HER and the MoS_2_-SiNWs-SWNTs composites for lithium-ion batteries offer significant potential as dual-function materials for sustainable energy applications. Their demonstrated efficiency in hydrogen production and energy storage underscores their versatility and value in the development of renewable energy technologies. Future efforts should aim to optimize these composites further, focusing on improving their long-term stability and scalability for practical, large-scale applications.

## Data Availability

The data is contained in the article and is available from the corresponding authors on reasonable request.

## Supplementary Materials

The following supporting information can be downloaded at: https://www.mdpi.com/article/10.3390/nano14231911/s1, Table S1. BET surface area of different composites; Table S2. Elemental composition of MoS_2_@SiNWs@CNT and MoS_2_@CNT electrodes after 100 charge–discharge cycles, measured by nuclear reaction analysis (NRA); Figure S1. Effect of pH on the photocatalytic activity of the 6.7% (MoS_2_-SiNWs)@ZnONPs-CNTs catalyst for hydrogen production; Figure S2. Evaluation of the initial concentration of the 6.7% (MoS_2_-SiNWs)@ZnONPs-CNTs catalyst on the efficiency of hydrogen production; Figure S3. Control experiments for 6.7% (MoS_2_-SiNWs)@ZnONPs-CNTs on the efficiency of hydrogen production; Figure S4. Hydrogen production via water splitting using 6.7% (MoS_2_-SiNWs)@ZnONPs-CNTs under irradiation, and also in the presence of a hole scavenger, namely EDTA-Na_2_; Figure S5. Recyclability of 6.7% (MoS_2_-SiNWs)@ZnONPs-CNTs after 10 consecutive catalytic cycles of hydrogen production, under irradiation at 500 nm; Figure S6. Photoluminescence of ZnONPs (a); ZnONPs-CNTs (b); 5% (MoS_2_-SiNWs)@ZnONPs-CNTs (c); and 6.7% (MoS_2_-SiNWs)@ZnONPs-CNTs (d), by a 350 nm excitation at room temperature; Figure S7. Differential capacity (dQ/dV) curves for (a) MoS_2_@CNT at 0.1 A/g, (b) MoS_2_@SiNW@CNT at 0.1 A/g, and (c) MoS_2_@SiNW@CNT at 1 A/g; Figure S8. Cross-sectional SEM images of pristine and cycled electrodes: (a) MoS_2_@CNT (80:20 wt.%) before cycling, (b) MoS_2_@CNT after 100 cycles at 1 A/g, (c) MoS_2_@SiNW@CNT (56:24:20 wt.%) before cycling, and (d) MoS_2_@SiNW@CNT after 100 cycles at 1 A/g.

## References

[1] Hassan Q., Viktor P., Al-Musawi T.J., Mahmood Ali B., Algburi S., Alzoubi H.M., Khudhair Al-Jiboory A., Zuhair Sameen A., Salman H.M., Jaszczur M. (2024). The Renewable Energy Role in the Global Energy Transformations. Renew. Energy Focus.

[2] (2024). World Economic Forum Fostering Effective Energy Transition. https://www.weforum.org/publications/fostering-effective-energy-transition-2024/in-full/3-sub-index-and-dimension-trends/.

[3] Simpa P., Solomon N.O., Adenekan O.A., Obasi S.C. (2024). Nanotechnology’s Potential in Advancing Renewable Energy Solutions. Eng. Sci. Technol. J..

[4] Wang P., Dong Q., Gao C., Bai W., Chu D., He Y. (2024). A Comprehensive Review of Carbon Nanotubes: Growth Mechanisms, Preparation and Applications. Fullerenes, Nanotubes and Carbon. Nanostructures.

[5] Hughes K.J., Iyer K.A., Bird R.E., Ivanov J., Banerjee S., Georges G., Zhou Q.A. (2024). Review of Carbon Nanotube Research and Development: Materials and Emerging Applications. ACS Appl. Nano Mater..

[6] Raman S., Sankar R., M S. (2023). Advances in Silicon Nanowire Applications in Energy Generation, Storage, Sensing, and Electronics: A Review. Nanotechnology.

[7] Srivastava T., Shrivastav A.M., Sinha S., Polley D., Alee K.S., Sujatha R.A., Mishra A., Saxena S.K., Mishra A., Dixit V., Somvanshi D., Singh A., Mishra A. (2024). Silicon Nanowire: From Fabrication to Its Application. Materials for Electronic, Magnetic, and Spintronic Technologies.

[8] Ingsel T., Gupta R.K. (2022). Nanostructured Silicon for Energy Applications. Silicon-Based Hybrid Nanoparticles.

[9] Samy O., El Moutaouakil A. (2021). A Review on MoS_2_ Energy Applications: Recent Developments and Challenges. Energies.

[10] Gunawan D., Zhang J., Li Q., Toe C.Y., Scott J., Antonietti M., Guo J., Amal R. (2024). Materials Advances in Photocatalytic Solar Hydrogen Production: Integrating Systems and Economics for a Sustainable Future. Adv. Mater..

[11] Singla S., Sharma S., Basu S., Shetti N.P., Aminabhavi T.M. (2021). Photocatalytic Water Splitting Hydrogen Production via Environmental Benign Carbon Based Nanomaterials. Int. J. Hydrogen Energy.

[12] Ganguly P., Harb M., Cao Z., Cavallo L., Breen A., Dervin S., Dionysiou D.D., Pillai S.C. (2019). 2D Nanomaterials for Photocatalytic Hydrogen Production. ACS Energy Lett..

[13] Gupta A., Likozar B., Jana R., Chanu W.C., Singh M.K. (2022). A Review of Hydrogen Production Processes by Photocatalytic Water Splitting—From Atomistic Catalysis Design to Optimal Reactor Engineering. Int. J. Hydrogen Energy.

[14] Chu X., Sathish C.I., Yang J., Guan X., Zhang X., Qiao L., Domen K., Wang S., Vinu A., Yi J. (2023). Strategies for Improving the Photocatalytic Hydrogen Evolution Reaction of Carbon Nitride-Based Catalysts. Small.

[15] Zhang Y., Heo Y.-J., Lee J.-W., Lee J.-H., Bajgai J., Lee K.-J., Park S.-J. (2018). Photocatalytic Hydrogen Evolution via Water Splitting: A Short Review. Catalysts.

[16] Molaei M.J. (2024). Recent Advances in Hydrogen Production through Photocatalytic Water Splitting: A Review. Fuel.

[17] Nazir M.A., Najam T., Altaf M., Ahmad K., Hossain I., Assiri M.A., Javed M.S., Rehman A.U., Shah S.S.A. (2024). Tuning the Photocatalytic Hydrogen Production via Co-Catalyst Engineering. J. Alloys Compd..

[18] Suman N., Irfan H., Shanmugharaj A.M., Mohamed Riyaz A.K., Pai R.K., Deshmukh K., Pandey M. (2024). Layered Transition Metal Dichalcogenide-Based Nanomaterials for Lithium-Ion Batteries. Nanostructured Materials for Energy Storage.

[19] Hossain M.H., Chowdhury M.A., Hossain N., Islam M.A., Mobarak M.H. (2023). Advances of Lithium-Ion Batteries Anode Materials—A Review. Chem. Eng. J. Adv..

[20] Dang L., Yuan Y., Wang Z., Li H., Yang R., Fu A., Liu X., Li H. (2023). Carbon Nanofibers Decorated by MoS_2_ Nanosheets with Tunable Quantity as Self-Supporting Anode for High-Performance Lithium Ion Batteries. Nanomaterials.

[21] Poorshakoor E., Darab M. (2024). Advancements in the Development of Nanomaterials for Lithium-Ion Batteries: A Scientometric Review. J. Energy Storage.

[22] Li W., Qian X., Hou S., Xia X., He D., Xia J., He G., Chen H. (2024). Recent Progress of Self-Supported Anode Materials for Li-Ion Batteries. J. Energy Storage.

[23] Shams S., Bindhu B. (2024). Recent Advancements in Hybrid Two Dimensional Materials for Energy Applications. ES Energy Environ..

[24] Sun J., Guo F., Ai X., Tian Y., Yang J., Zou X., Zhu G. (2024). Constructing Heterogeneous Interface by Growth of Carbon Nanotubes on the Surface of MoB_2_ for Boosting Hydrogen Evolution Reaction in a Wide pH Range. Small.

[25] Kim J.H., Kim S., Han J.H., Seo S.B., Choi Y.R., Lim J., Kim Y.A. (2023). Perspective on Carbon Nanotubes as Conducting Agent in Lithium-Ion Batteries: The Status and Future Challenges. Carbon Lett..

[26] Sehrawat P., Julien C., Islam S.S. (2016). Carbon Nanotubes in Li-Ion Batteries: A Review. Mater. Sci. Eng. B.

[27] Chen X., Bi Q., Sajjad M., Wang X., Ren Y., Zhou X., Xu W., Liu Z. (2018). One-Dimensional Porous Silicon Nanowires with Large Surface Area for Fast Charge–Discharge Lithium-Ion Batteries. Nanomaterials.

[28] Ray U., Sarkar S., Banerjee D. (2023). Silicon Nanowires as an Efficient Material for Hydrogen Evolution through Catalysis: A Review. Catal. Today.

[29] Yang C., Chandran K.S.R. (2023). A Critical Review of Silicon Nanowire Electrodes and Their Energy Storage Capacities in Li-Ion Cells. RSC Adv..

[30] Li G., Zhang D., Qiao Q., Yu Y., Peterson D., Zafar A., Kumar R., Curtarolo S., Hunte F., Shannon S. (2016). All The Catalytic Active Sites of MoS_2_ for Hydrogen Evolution. J. Am. Chem. Soc..

[31] Wang J., Lu L., Lotya M., Coleman J.N., Chou S., Liu H., Minett A.I., Chen J. (2013). Development of MoS_2_–CNT Composite Thin Film from Layered MoS_2_ for Lithium Batteries. Adv. Energy Mater..

[32] Machín A., Cotto M., Duconge J., Arango J.C., Morant C., Pinilla S., Soto-Vázquez L., Resto E., Márquez F. (2018). Hydrogen Production via Water Splitting Using Different Au@ZnO Catalysts under UV–Vis Irradiation. J. Photochem. Photobiol. A Chem..

[33] Ghorai A., Ray S.K., Midya A. (2019). Ethylenediamine-Assisted High Yield Exfoliation of MoS_2_ for Flexible Solid-State Supercapacitor Application. ACS Appl. Nano Mater..

[34] Redondo-Cubero A., Borge M.J.G., Gordillo N., Gutiérrez P.C., Olivares J., Pérez Casero R., Ynsa M.D. (2021). Current Status and Future Developments of the Ion Beam Facility at the Centre of Micro-Analysis of Materials in Madrid. Eur. Phys. J. Plus.

[35] Mathayan V., Moro M.V., Morita K., Tsuchiya B., Ye R., Baba M., Primetzhofer D. (2020). In-Operando Observation of Li Depth Distribution and Li Transport in Thin Film Li Ion Batteries. Appl. Phys. Lett..

[36] Mathayan V., Morita K., Tsuchiya B., Ye R., Baba M., Moro M.V., Primetzhofer D. (2021). Assessing the Potential of Ion Beam Analytical Techniques for Depth Profiling Li in Thin Film Li Ion Batteries. J. Appl. Phys..

[37] Paneta V., Kafkarkou A., Kokkoris M., Lagoyannis A. (2012). Differential Cross-Section Measurements for the ^7^Li(p,p_0_)^7^Li, ^7^Li(p,p_1_)^7^Li, ^7^Li(p,α_0_)^4^He, ^19^F(p,p_0_)^19^F, ^19^F(p,α_0_)^16^O and ^19^F(p,α_1,2_)^16^O Reactions. Nucl. Instrum. Methods Phys. Res. Sect. B Beam Interact. Mater. At..

[38] Mayer M. (1997). SIMNRA User’s Guide.

[39] Mayer M. (2002). Ion Beam Analysis of Rough Thin Films. Nucl. Instrum. Methods Phys. Res. Sect. B Beam Interact. Mater. At..

[40] Mayer M. (1999). SIMNRA, a Simulation Program for the Analysis of NRA, RBS and ERDA. AIP Conference Proceedings.

[41] Baruah S., Thanachayanont C., Dutta J. (2008). Growth of ZnO Nanowires on Nonwoven Polyethylene Fibers. Sci. Technol. Adv. Mater..

[42] Miralrio A., Rangel Cortes E., Castro M. (2018). Electronic Properties and Enhanced Reactivity of MoS_2_ Monolayers with Substitutional Gold Atoms Embedded into Sulfur Vacancies. Appl. Surf. Sci..

[43] Dresselhaus M.S., Jorio A., Hofmann M., Dresselhaus G., Saito R. (2010). Perspectives on Carbon Nanotubes and Graphene Raman Spectroscopy. Nano Lett..

[44] Iatsunskyi I., Nowaczyk G., Jurga S., Fedorenko V., Pavlenko M., Smyntyna V. (2015). One and Two-Phonon Raman Scattering from Nanostructured Silicon. Optik.

[45] Mottola S., Mancuso A., Sacco O., Vaiano V., De Marco I. (2023). Photocatalytic Systems Based on ZnO Produced by Supercritical Antisolvent for Ceftriaxone Degradation. Catalysts.

[46] Cuscó R., Alarcón-Lladó E., Ibáñez J., Artús L., Jiménez J., Wang B., Callahan M.J. (2007). Temperature Dependence of Raman Scattering in ZnO. Phys. Rev. B.

[47] Sharma A., Singh B.P., Dhar S., Gondorf A., Spasova M. (2012). Effect of Surface Groups on the Luminescence Property of ZnO Nanoparticles Synthesized by Sol–Gel Route. Surf. Sci..

[48] Fontánez K., García D., Ortiz D., Sampayo P., Hernández L., Cotto M., Ducongé J., Díaz F., Morant C., Petrescu F. (2022). Biomimetic Catalysts Based on Au@TiO_2_-MoS_2_-CeO_2_ Composites for the Production of Hydrogen by Water Splitting. Int. J. Mol. Sci..

[49] Castellanos-Gomez A., Quereda J., van der Meulen H.P., Agraït N., Rubio-Bollinger G. (2016). Spatially Resolved Optical Absorption Spectroscopy of Single- and Few-Layer MoS_2_ by Hyperspectral Imaging. Nanotechnology.

[50] Li H., Zhang Q., Yap C.C.R., Tay B.K., Edwin T.H.T., Olivier A., Baillargeat D. (2012). From Bulk to Monolayer MoS_2_: Evolution of Raman Scattering. Adv. Funct. Mater..

[51] Ahmad M., Rehman W., Khan M.M., Qureshi M.T., Gul A., Haq S., Ullah R., Rab A., Menaa F. (2021). Phytogenic Fabrication of ZnO and Gold Decorated ZnO Nanoparticles for Photocatalytic Degradation of Rhodamine B. J. Environ. Chem. Eng..

[52] Ngoma M.M., Mathaba M., Moothi K. (2021). Effect of Carbon Nanotubes Loading and Pressure on the Performance of a Polyethersulfone (PES)/Carbon Nanotubes (CNT) Membrane. Sci. Rep..

[53] Al-Taay H.F., Mahdi M.A., Parlevliet D., Jennings P. (2017). Fabrication and Characterization of Solar Cells Based on Silicon Nanowire Homojunctions. Silicon.

[54] Ghasemipour P., Fattahi M., Rasekh B., Yazdian F. (2020). Developing the Ternary ZnO Doped MoS_2_ Nanostructures Grafted on CNT and Reduced Graphene Oxide (RGO) for Photocatalytic Degradation of Aniline. Sci. Rep..

[55] Ren B., Shen W., Li L., Wu S., Wang W. (2018). 3D CoFe_2_O_4_ Nanorod/Flower-like MoS_2_ Nanosheet Heterojunctions as Recyclable Visible Light-Driven Photocatalysts for the Degradation of Organic Dyes. Appl. Surf. Sci..

[56] Briggs D., Seah M. (1994). Practical Surface Analysis.

[57] Lee H.-J., Kim J.S., Lee K.Y., Park K.H., Bae J.-S., Mubarak M., Lee H. (2019). Elucidation of an Intrinsic Parameter for Evaluating the Electrical Quality of Graphene Flakes. Sci. Rep..

[58] Kim B.-J., Kim J.-P., Park J.-S. (2014). Effects of Al Interlayer Coating and Thermal Treatment on Electron Emission Characteristics of Carbon Nanotubes Deposited by Electrophoretic Method. Nanoscale Res. Lett..

[59] Tan S.M., Ambrosi A., Chua C.K., Pumera M. (2014). Electron Transfer Properties of Chemically Reduced Graphene Materials with Different Oxygen Contents. J. Mater. Chem. A.

[60] Morimoto N., Kubo T., Nishina Y. (2016). Tailoring the Oxygen Content of Graphite and Reduced Graphene Oxide for Specific Applications. Sci. Rep..

[61] Zagorac D., Zagorac J., Pejić M., Matović B., Schön J.C. (2022). Band Gap Engineering of Newly Discovered ZnO/ZnS Polytypic Nanomaterials. Nanomaterials.

[62] Hanif M.A., Kim Y.-S., Akter J., Kim H.G., Kwac L.K. (2023). Fabrication of Robust and Stable N-Doped ZnO/Single-Walled Carbon Nanotubes: Characterization, Photocatalytic Application, Kinetics, Degradation Products, and Toxicity Analysis. ACS Omega.

[63] Rahman I.A., Purqon A. (2017). First Principles Study of Molybdenum Disulfide Electronic Structure. J. Phys. Conf. Ser..

[64] Hutagalung S.D., Fadhali M.M., Areshi R.A., Tan F.D. (2017). Optical and Electrical Characteristics of Silicon Nanowires Prepared by Electroless Etching. Nanoscale Res. Lett..

[65] Prabavathi S.L., Saravanakumar K., Nkambule T.T.I., Muthuraj V., Mamba G. (2020). Enhanced Photoactivity of Cerium Tungstate-Modified Graphitic Carbon Nitride Heterojunction Photocatalyst for the Photodegradation of Moxifloxacin. J. Mater. Sci. Mater. Electron..

[66] Jourshabani M., Shariatinia Z., Badiei A. (2018). Synthesis and Characterization of Novel Sm_2_O_3_/S-Doped g-C_3_N_4_ Nanocomposites with Enhanced Photocatalytic Activities under Visible Light Irradiation. Appl. Surf. Sci..

[67] Cao J., Li X., Lin H., Chen S., Fu X. (2012). In Situ Preparation of Novel p–n Junction Photocatalyst BiOI/(BiO)_2_CO_3_ with Enhanced Visible Light Photocatalytic Activity. J. Hazard. Mater..

[68] Chen C., Bi W., Xia Z., Yuan W., Li L. (2020). Hydrothermal Synthesis of the CuWO_4_/ZnO Composites with Enhanced Photocatalytic Performance. ACS Omega.

[69] Barpuzary D., Banik A., Gogoi G., Qureshi M. (2015). Noble Metal-Free Counter Electrodes Utilizing Cu_2_ZnSnS_4_ Loaded with MoS_2_ for Efficient Solar Cells Based on ZnO Nanowires Co-Sensitized with CuInS_2_–CdSe Quantum Dots. J. Mater. Chem. A.

[70] Zhang Y., Mandal R., Ratchford D.C., Anthony R., Yeom J. (2020). Si Nanocrystals/ZnO Nanowires Hybrid Structures as Immobilized Photocatalysts for Photodegradation. Nanomaterials.

[71] Zhu Y., Ling Q., Liu Y., Wang H., Zhu Y. (2015). Photocatalytic H_2_ Evolution on MoS_2_–TiO_2_ Catalysts Synthesized via Mechanochemistry. Phys. Chem. Chem. Phys..

[72] Liu Y., Xu X., Zhang J., Zhang H., Tian W., Li X., Tade M.O., Sun H., Wang S. (2018). Flower-like MoS_2_ on Graphitic Carbon Nitride for Enhanced Photocatalytic and Electrochemical Hydrogen Evolutions. Appl. Catal. B Environ..

[73] Dong J., Fang W., Yuan H., Xia W., Zeng X., Shangguan W. (2022). Few-Layered MoS_2_/ZnCdS/ZnS Heterostructures with an Enhanced Photocatalytic Hydrogen Evolution. ACS Appl. Energy Mater..

[74] Zeimpekis I., Rahman T., Leung O.M., Tyson J., Ebert M., Boden S.A., Ponce De Leon C., Morgan K.A. (2024). Scalable Large-Area 2D-MoS_2_/Silicon-Nanowire Heterostructures for Enhancing Energy Storage Applications. ACS Appl. Energy Mater..

[75] Stoica I., Abraham A.R., Haghi A.K. (2023). Advances in Energy Materials: New Composites and Techniques for Future Energy Applications.

[76] Anushya G., Benjamin M., Sarika R., Pravin J.C., Sridevi R., Nirmal D. (2024). A Review on Applications of Molybdenum Disulfide Material: Recent Developments. Micro Nanostruct..

[77] Fang X., Hua C., Guo X., Hu Y., Wang Z., Gao X., Wu F., Wang J., Chen L. (2012). Lithium Storage in Commercial MoS_2_ in Different Potential Ranges. Electrochim. Acta.

[78] Ma L., Ye J., Chen W., Chen D., Yang Lee J. (2014). Gemini Surfactant Assisted Hydrothermal Synthesis of Nanotile-like MoS_2_/Graphene Hybrid with Enhanced Lithium Storage Performance. Nano Energy.

[79] Chen B., Meng Y., He F., Liu E., Shi C., He C., Ma L., Li Q., Li J., Zhao N. (2017). Thermal Decomposition-Reduced Layer-by-Layer Nitrogen-Doped Graphene/MoS_2_/Nitrogen-Doped Graphene Heterostructure for Promising Lithium-Ion Batteries. Nano Energy.

[80] Cheng Y., Lu S., Zhang H., Varanasi C.V., Liu J. (2012). Synergistic Effects from Graphene and Carbon Nanotubes Enable Flexible and Robust Electrodes for High-Performance Supercapacitors. Nano Lett..

[81] Wang Y., Yu L., Lou X.W. (2016). (David) Synthesis of Highly Uniform Molybdenum–Glycerate Spheres and Their Conversion into Hierarchical MoS_2_ Hollow Nanospheres for Lithium-Ion Batteries. Angew. Chem. Int. Ed..

[82] Kong X., Xi Z., Wang L., Zhou Y., Liu Y., Wang L., Li S., Chen X., Wan Z. (2023). Recent Progress in Silicon−Based Materials for Performance−Enhanced Lithium−Ion Batteries. Molecules.

[83] Chang K., Chen W. (2011). L-Cysteine-Assisted Synthesis of Layered MoS_2_/Graphene Composites with Excellent Electrochemical Performances for Lithium Ion Batteries. ACS Nano.

[84] Shi Y., Wang Y., Wong J.I., Tan A.Y.S., Hsu C.-L., Li L.-J., Lu Y.-C., Yang H.Y. (2013). Self-Assembly of Hierarchical MoS_x_/CNT Nanocomposites (2 < x < 3): Towards High Performance Anode Materials for Lithium Ion Batteries. Sci. Rep..

[85] Gauthier M. (2013). Electrodes Negatives a Base de Silicium Pour Accumulateurs Au Lithium: Mecanisme Reactionnel a L’echelle Nanometrique et Optimisation Des Performances. Ph.D. Thesis.

[86] Obrovac M.N., Chevrier V.L. (2014). Alloy Negative Electrodes for Li-Ion Batteries. Chem. Rev..

[87] Chhowalla M., Shin H.S., Eda G., Li L.-J., Loh K.P., Zhang H. (2013). The Chemistry of Two-Dimensional Layered Transition Metal Dichalcogenide Nanosheets. Nat. Chem..

[88] Beaulieu L.Y., Eberman K.W., Turner R.L., Krause L.J., Dahn J.R. (2001). Colossal Reversible Volume Changes in Lithium Alloys. Electrochem. Solid-State Lett..

[89] Xiu Z., Kim D., Alfaruqi M.H., Song J., Kim S., Duong P.T., Mathew V., Baboo J.P., Kim J. (2017). Ultrafine Molybdenum Oxycarbide Nanoparticles Embedded in N-Doped Carbon as a Superior Anode Material for Lithium-Ion Batteries. J. Alloys Compd..

[90] Obrovac M.N., Christensen L. (2004). Structural Changes in Silicon Anodes during Lithium Insertion/Extraction. Electrochem. Solid-State Lett..

[91] Pinilla S., Park S.-H., Fontanez K., Márquez F., Nicolosi V., Morant C. (2020). 0D-1D Hybrid Silicon Nanocomposite as Lithium-Ion Batteries Anodes. Nanomaterials.

