# Supplementary material for: Hydrogen Production and Li-Ion Battery Performance with MoS2-SiNWs-SWNTs@ZnONPs Nanocomposites
Source: Nanomaterials (Basel). 2024 Nov 28;14(23):1911. doi: 10.3390/nano14231911 (PMC11643952; doi:10.3390/nano14231911)
Supplement: Supplementary file 1 [file nanomaterials-14-01911-s001.zip › nanomaterials-3263280-supplementary.pdf]

nanomaterials

Supplementary Materials

Hydrogen Production and Li-ion Battery Performance with MoS_2-SiNWs-SWNTs@ZnONPs Nanocomposites

Abniel Machín[1], María C. Cotto[2], Francisco Márquez*[2], Jesús Díaz-Sánchez[3,4], Celia Polop[3,4,5], Carmen Morant*[4,6]

[1] Division of Natural Sciences and Technology, Universidad Ana G. Méndez-Cupey Campus, San Juan, PR 00926, USA; machina1@uagm.edu
[2] Nanomaterials Research Group, School of Natural Sciences and Technology, Universidad Ana G. Méndez-Gurabo Campus, Gurabo, PR 00778, USA; mcotto48@uagm.edu
[3] Department of Condensed Matter Physics, Universidad Autónoma de Madrid, 28049 Madrid, Spain; jesus.diazs@uam.es (J.D.-S.); celia.polop@uam.es (C.P.)
[4] Instituto de Ciencia de Materiales Nicolás Cabrera, Universidad Autónoma de Madrid, 28049 Madrid, Spain
[5] Condensed Matter Physics Center (IFIMAC), Universidad Autónoma de Madrid, 28049 Madrid, Spain
[6] Department of Applied Physics, Universidad Autónoma de Madrid, 28049 Madrid, Spain
* Correspondence: fmarquez@uagm.edu (F.M.); c.morant@uam.es (C.M.); Tel.: +1-787-743-7979 (ext. 4250) (F.M.); +34-914974924 (C.M.)

Table S1. BET surface area of the different composites.

Catalyst	BET surface area (m^2g^{-1})
ZnONPs	62
ZnONPs-SiNWs-CNTs	297
5%(MoS_2-SiNWs)@ZnONPs-CNTs	231
6.7%(MoS_2-SiNWs)@ZnONPs-CNTs	246

Table S2. Elemental composition of MoS_2@SiNWs@CNT and MoS_2@CNT electrodes after 100 charge/discharge cycles, measured by Nuclear Reaction Analysis (NRA).

Atomic %	Li	C	O	F	Si	S	Mo
MoS_2@CNTs	22.5	24.3 (excess 16.9)	21.1	25.3		4.5	2.2
MoS_2@SiNW@CNTs	28.9	21.7 (excess 5.1)	35.0		5.8	5.8	2.9

Film excess mass (%)	Li	C	O	F
MoS_2@CNTs	35.1	45.2	75.1	107.2
MoS_2@SiNW@CNTs	27.5	8.4	76.7	

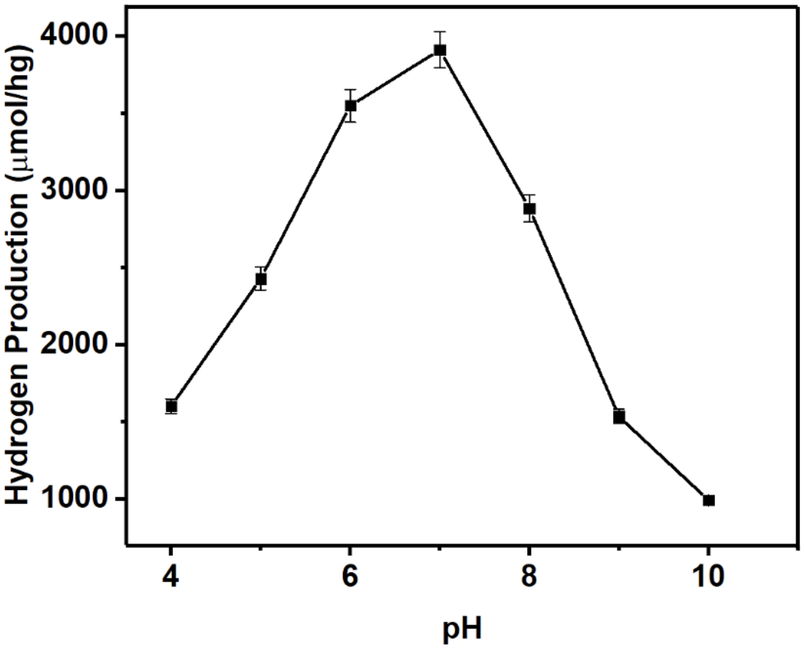

Figure S1. Effect of pH on the photocatalytic activity of the 6.7%(MoS_2-SiNWs)@ZnONPs-CNTs catalyst for hydrogen production.

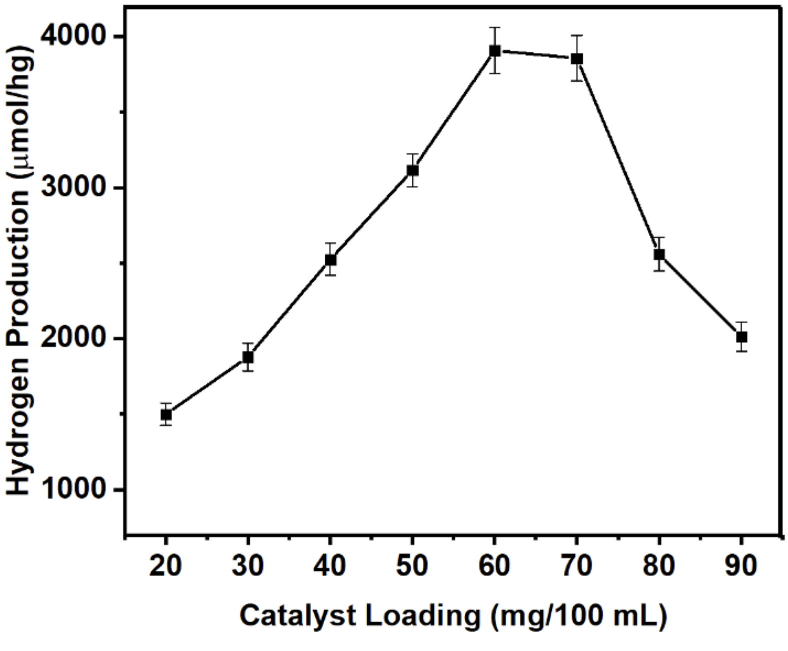

Figure S2. Evaluation of the initial concentration of the 6.7%(MoS_2-SiNWs)@ZnONPs-CNTs catalyst on the efficiency of hydrogen production.

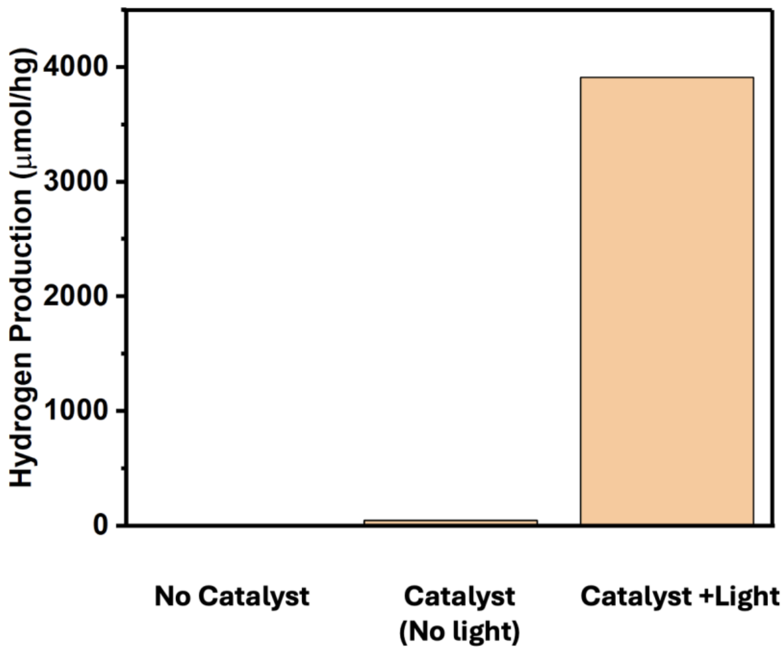

Figure S3. Control experiments for 6.7%(MoS_2-SiNWs)@ZnONPs-CNTs on the efficiency of hydrogen production.

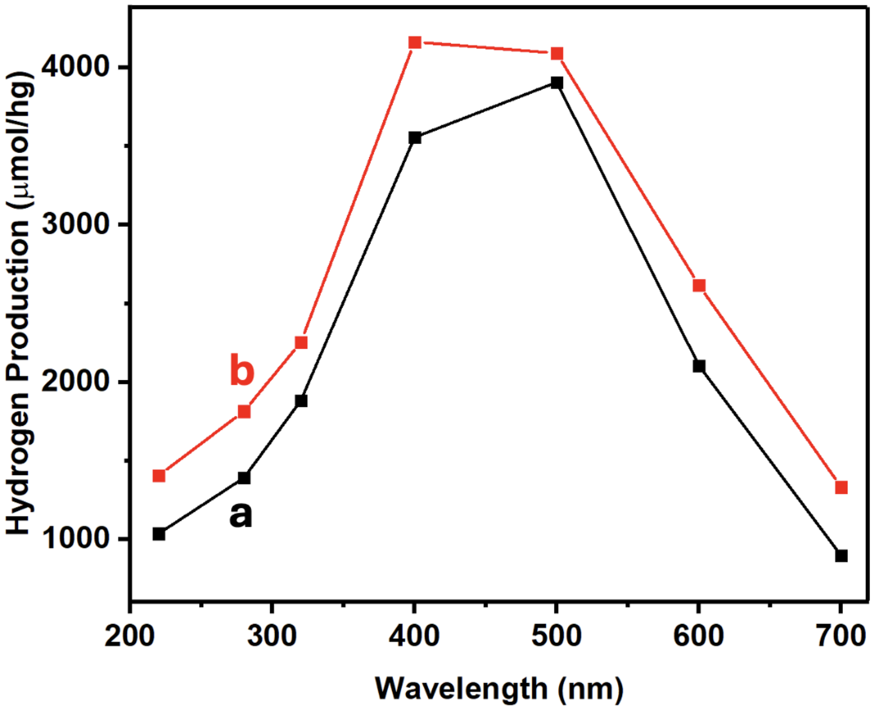

Figure S4. Hydrogen production via water splitting using 6.7%(MoS_2-SiNWs)@ZnONPs-CNTs under irradiation (a), and also in the presence of a hole scavenger, namely EDTA-Na_2 (b).

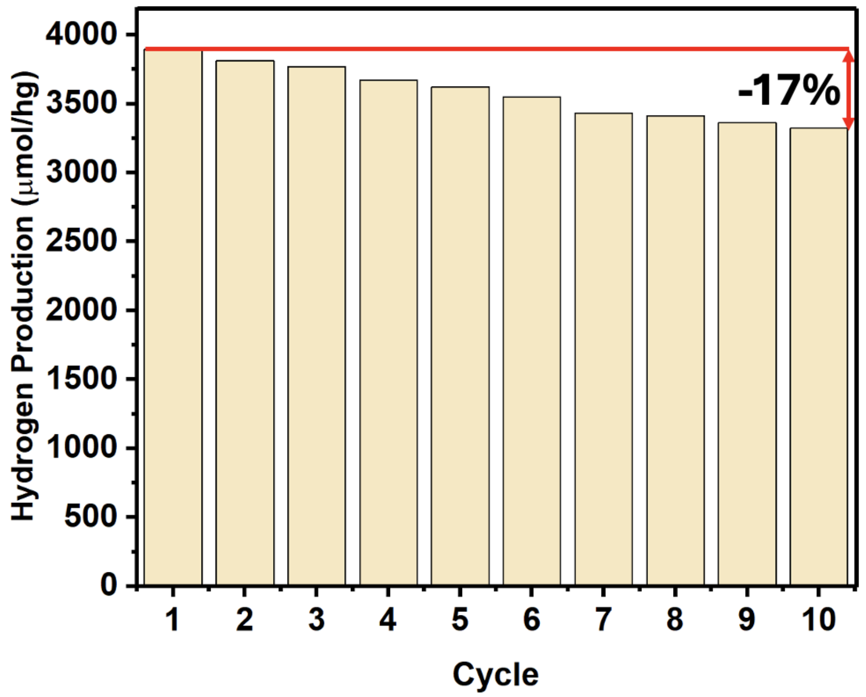

Figure S5. Recyclability of 6.7%(MoS_2-SiNWs)@ZnONPs-CNTs after 10 consecutive catalytic cycles of hydrogen production, under irradiation at 500 nm.

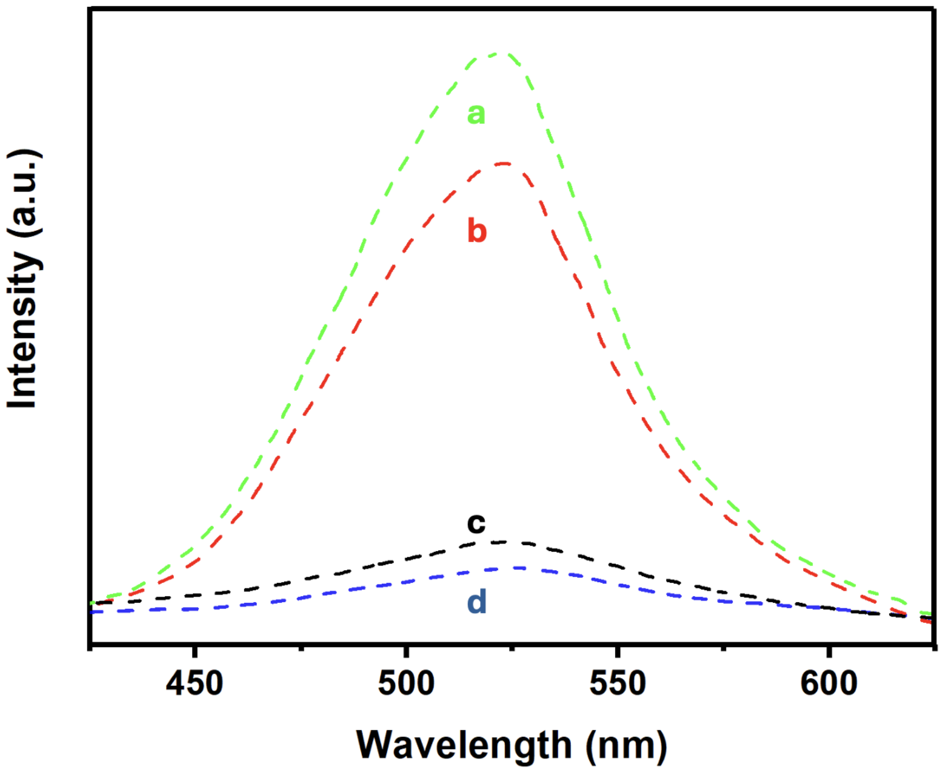

Figure S6. Photoluminescence of ZnONPs (a); ZnONPs-CNTs (b); 5%(MoS_2-SiNWs)@ZnONPs-CNTs (c); and 6.7%(MoS_2-SiNWs)@ZnONPs-CNTs (d), by a 350 nm excitation at room temperature.

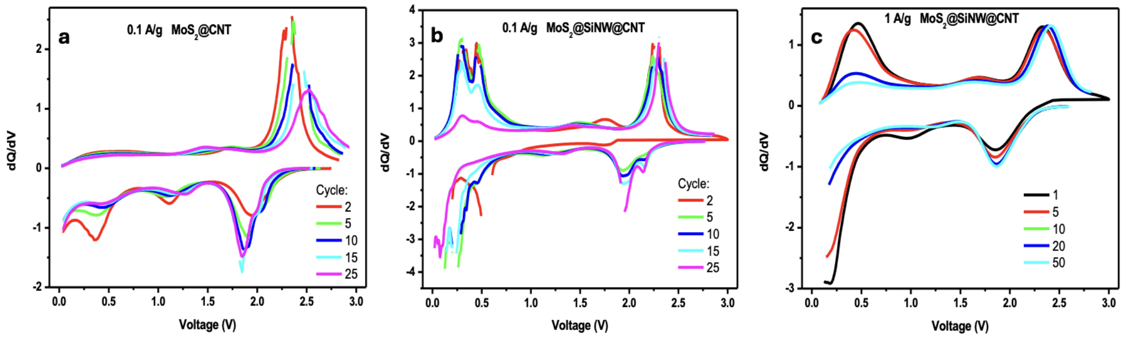

Figure S7. Differential capacity (dQ/dV) curves for (a) MoS_2@CNT at 0.1 A/g, (b) MoS_2@SiNW@CNT at 0.1 A/g, and (c) MoS_2@SiNW@CNT at 1 A/g.

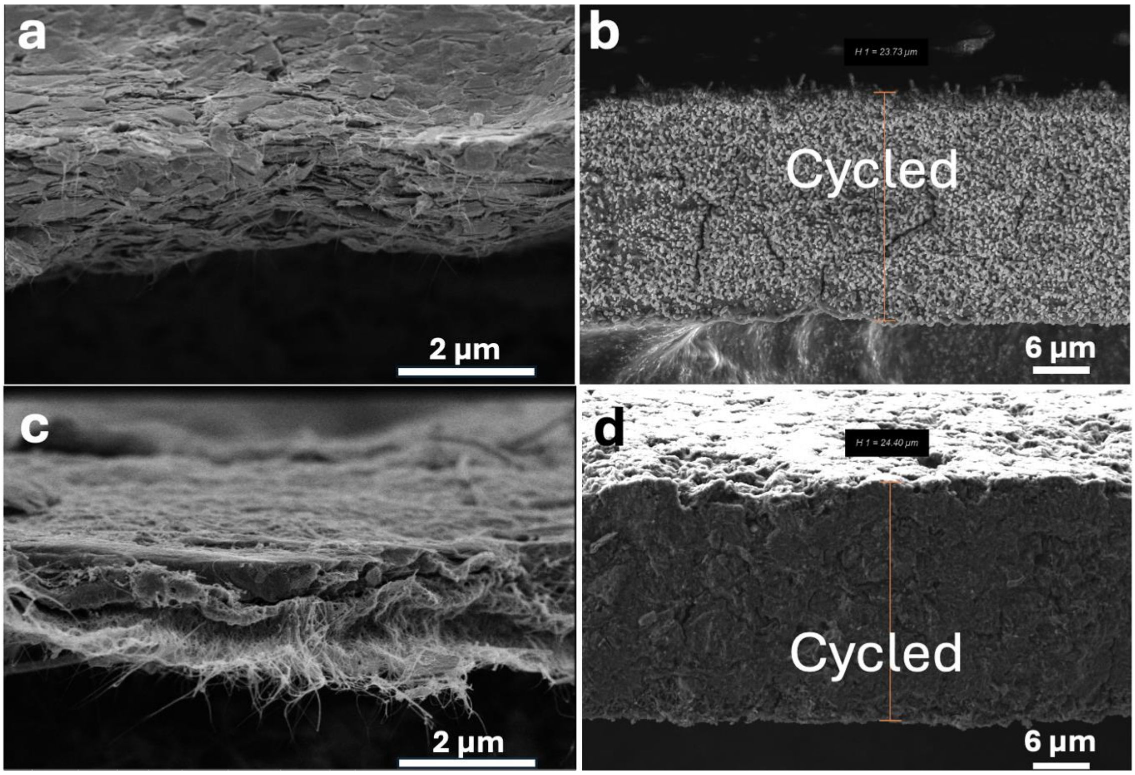

Figure S8. Cross-sectional SEM images of pristine and cycled electrodes: (a) MoS_2@CNT (80:20 wt.%) before cycling, (b) MoS_2@CNT after 100 cycles at 1 A/g, (c) MoS_2@SiNW@CNT (56:24:20 wt.%) before cycling, and (d) MoS_2@SiNW@CNT after 100 cycles at 1 A/g. .
